# Generation of transgene-free *PDS* mutants in potato by *Agrobacterium*-mediated transformation

**DOI:** 10.1186/s12896-020-00621-2

**Published:** 2020-05-12

**Authors:** Zsófia Bánfalvi, Edina Csákvári, Vanda Villányi, Mihály Kondrák

**Affiliations:** NARIC Agricultural Biotechnology Institute, H-2100 Szent-Györgyi A. u. 4., Gödöllő, Hungary

**Keywords:** *Agrobacterium*, Chimaera, CRISPR/Cas9, Gene editing, Potato, Transgene-free, Transformation

## Abstract

**Background:**

Gene editing using the CRISPR/Cas9 system has become a routinely applied method in several plant species. The most convenient gene delivery system is *Agrobacterium*-mediated gene transfer with antibiotic selection and stable genomic integration of transgenes, including *Cas9*. For elimination of transgenes in the segregating progeny, selfing is applied in many plant species. This approach, however, cannot be widely employed in potato because most of the commercial potato cultivars are self-incompatible.

**Results:**

In this study, the efficiency of a transient *Cas9* expression system with positive/negative selection based on *codA-nptII* fusion was tested. The *PHYTOENE DESATURASE* (*PDS*) gene involved in carotenoid biosynthesis was targeted. A new vector designated PROGED::gPDS carrying only the right border of T-DNA was constructed. Using only the positive selection function of PROGED::gPDS and the restriction enzyme site loss method in PCR of genomic DNA after digestion with the appropriate restriction enzyme, it was demonstrated that the new vector is as efficient in gene editing as a traditional binary vector with right- and left-border sequences. Nevertheless, 2 weeks of positive selection followed by negative selection did not result in the isolation of *PDS* mutants. In contrast, we found that with 3-day positive selection, *PDS* mutants appear in the regenerating population with a minimum frequency of 2–10%. Interestingly, while large deletions (> 100 bp) were generated by continuous positive selection, the 3-day selection resulted in deletions and substitutions of only a few bp. Two albinos and three chimaeras with white and green leaf areas were found among the *PDS* mutants, while all the other *PDS* mutant plants were green. Based on DNA sequence analysis some of the green plants were also chimaeras. Upon vegetative propagation from stem segments in vitro*,* the phenotype of the plants obtained even by positive selection did not change, suggesting that the expression of *Cas9* and *gPDS* is silenced or that the DNA repair system is highly active during the vegetative growth phase in potato.

**Conclusions:**

Gene-edited plants can be obtained from potatoes by *Agrobacterium*-mediated transformation with 3-day antibiotic selection with a frequency high enough to identify the mutants in the regenerating plant population using PCR.

## Background

Genome editing strategies based on the Clustered Regulatory Interspaced Short Palindromic Repeat (CRISPR)-Associated Protein System (CRISPR/Cas9) were first shown to be effective in bacteria and mammalian cell lines, but they have been rapidly adapted for plant genome modification [1]. The CRISPR/Cas9 system has been used successfully in several food crops, including the most important Solanaceae species, tomato and potato [2, 3].

The first reports on successful CRISPR/Cas9-based genome editing in potato (*Solanum tuberosum* L.) were published in 2015. Wang et al. [4] cloned the native *U6* RNA promoter from potato to drive an oligo encoding single-guide RNA (sgRNA), which then acts as a guide to the specific site of the genome where Cas9 is able to cleave double-stranded DNA, leading to deletion, insertion or substitution at the target site. The *Cas9* used was derived from *Streptococcus pyogenes* and codon-optimised for rice [5]. It was driven by the CaMV *35S* promoter and cloned with the *U6* promoter::*sgRNA* into the binary vector pCAMBIA2300 and designated CP025. To test whether this CRISPR/Cas9 construct can introduce gene knockouts via *Agrobacterium*-mediated transformation, the *IAA2* encoding an auxin/indole-3-acetic acid family member protein was targeted in the double-haploid DM potato. Mono- and biallelic homozygous mutants, as well as heterozygous plants, were obtained without off-target mutations, demonstrating that the CRISPR/Cas9 system can be used for targeted mutagenesis in potato.

Butler et al. [6] targeted the *ACETOLACTATE SYNTHASE1* (*ALS1*) gene in a diploid breeding line and the tetraploid potato cultivar ‘Désirée’ using *Agrobacterium*-mediated transformation with either a conventional T-DNA or a modified geminivirus T-DNA. Both constructs were capable of generating targeted mutations leading to a reduced herbicide susceptibility phenotype. Single targeted mutations in primary events were capable of being carried through clonal generations and inherited through the germline as *Cas9*-free progeny. Later, it was demonstrated that with geminivirus replicons, even point mutations can be generated in potatoes [7].

The majority of potato cultivars possess gametophytic self-incompatibility. Thus, in most cases, selfing is not an applicable method to eliminate foreign genes and DNA fragments from the potato genome. Out-crossing also cannot be a good strategy, since potato is highly heterozygous. Thus, important traits would be lost in the next generation, which could be retained only by several back-crosses [8]. To avoid this problem, transient transfection and regeneration of protoplasts was used, or CRISPR-Cas9 ribonucleoproteins (RNPs) were delivered into protoplasts to alter the quality of starch by full knockout of the *GRANULE-BOUND STARCH SYNTHASE* (*GBSS*) gene [9, 10]. In 2–3% of the regenerated shoots from the RNP experiments, mutations were induced in all four alleles, resulting in a complete knockout of the GBSS enzyme function [10]. It was shown, however, that regeneration of potato plants from protoplasts induces widespread genome instability [11]. Furthermore, the regeneration method from protoplasts is not developed for a wide range of potato cultivars.

The most convenient gene delivery system for most crops is based on *Agrobacterium*-mediated gene transfer [12]. Chen et al. [13] reported a method for using *Agrobacterium* to transiently express *Cas9* and *sgRNA* in plant cells, using tobacco as a model plant and *PHYTOENE DESATURASE* (*PDS*) involved in carotenoid biosynthesis as a model target gene. However, a high-throughput screening protocol utilising next-generation sequencing in combination with high-resolution DNA melting (HRM) analysis was needed to efficiently identify mutants from a population of shoots regenerated in the absence of selection pressure. Recently, transgene-free tomato and potato mutants were successfully isolated by *Agrobacterium*-mediated delivery of a CRISPR/Cas9 cytidine base editor [14]. In this experiment, the *ALS* genes of potato were targeted, and after 2 weeks of kanamycin (Km) selection pressure, plant tissues were transferred to a selective medium containing chlorsulfuron for selection of *ALS* mutants. It was found that 10% of the mutants were transgene-free. Nevertheless, in the case of most of the target genes, there is no choice for the selection of edited plants.

In this paper, we report the results of testing the efficiency of a transient *Agrobacterium*-mediated genome-editing system by targeting the *PDS* gene in the cultivar ‘Désirée’ after two different intervals of Km selection followed either by lack of selection pressure or counter-selection for regeneration of plants with integrated transgenes. We demonstrate that while no transgene-free mutants could be obtained after 2 weeks of positive selection followed by negative selection, Km selection for 3 days resulted in the generation of transgene-free *PDS* mutants with a minimum frequency of 2–10%.

## Results

### Construction and testing the efficiency of a novel vector for gene editing

**Fig. 1:**
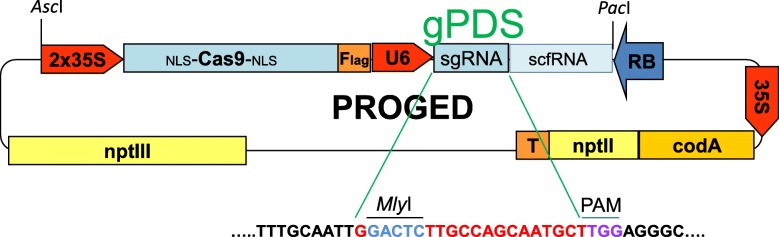
Schematic drawing of the PROGED vector with the *gPDS* coding sequence and the adjacent PAM motive that is essential for cleavage by Cas9 nuclease. Plant promoter regions are red-coloured. *RB*, right border of T-DNA; *nptIII*, *neomycin phosphotransferase III* with a bacterial promoter providing Km resistance for bacteria; *codA-nptII* encodes the fusion protein suitable for positive/negative (Km/5-FC) selection of gene-edited mutants after plant transformation

We previously reported that a binary vector, designated PROGMO, utilising an *R/Rs* recombinase system and a *codA-nptII* bi-functional, positive/negative selectable marker gene was successfully used for the generation of marker- and backbone-free transgenic potato plants [15]. PROGMO carries only the right border (*RB*) of T-DNA, and consequently, the whole plasmid will be inserted as one long T-DNA into the plant genome. This plasmid was converted to a vector suitable for gene editing. The recombinase gene was removed and replaced by the *Cas9-sgRNA-scaffoldRNA*-coding fragment derived from CP025 [4]. The novel vector was designated PROGED (Fig. 1).

To test the gene-editing efficiency of PROGED, *PDS* was selected as a target gene expecting the visible albino phenotype of *PDS* null mutants. The *PDS* gene of tomato cultivar ‘Micro Tom’ was edited by Pan et al. [16] using two different 20-bp *PDS* regions. One of them, *sgRNA2* designed to exon7, carried an *Mly*I restriction site. This provided the possibility of easy detection of mutations at the targeted locus by the restriction enzyme site loss method using PCR of genomic DNA after digestion with *Mly*I. To see how conserved this locus is in the potato cultivar ‘Désirée’, primers suitable for amplification of an approximately 550-bp region embodying the 20-bp *PDS* region were designed (Additional file 1: Table S1) and the targeted region amplified from genomic DNA. The PCR product was cloned into pGEM-T Easy and 10 clones were Sanger-sequenced. The 20-bp region had identical sequences in each clone with the corresponding tomato sequence (Additional file 2: Fig. S1). Thus, the 20-bp oligo was inserted into CP025 and moved into PROGED, resulting in PROGED::gPDS (Fig. 1).

**Fig. 2:**
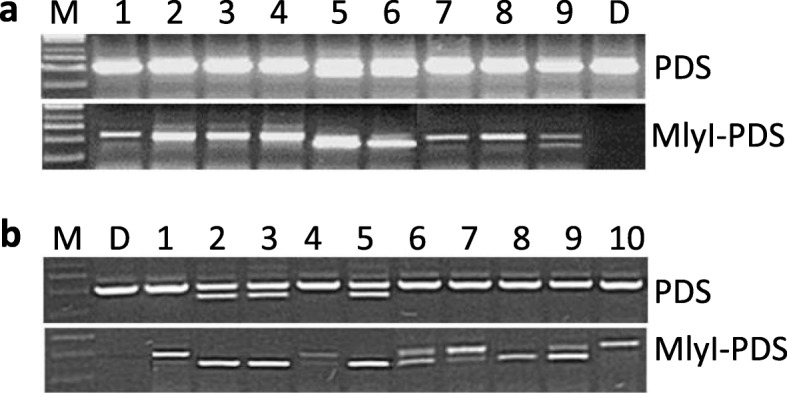
Detection of target mutations obtained with continuous Km selection in the *PDS* gene of potatoes by PCR assay using the primer pair PDS Fw and PDS R (Additional file 1: Table S1). **a**, 1–4, Detection of mutations in plants derived from tuber transformation with *A. tumefiaciens* LBA4404 (PROGED::gPDS); 5–9, detection of mutations in plants derived from tuber transformation with *A. tumefiaciens* gv2260 (PROGED::gPDS). Lane 9 shows the mutant PDS.5 m, the phenotype of which is shown in Fig. 3b. PCR was carried out with 36 cycles and fragments were separated on a 1% agarose gel. **b**, 1–5, Detection of mutations in plants derived from leaf transformation with *A. tumefiaciens* LBA4404 (PROGED::gPDS); 5–10, detection of mutations in plants derived from leaf transformation with *A. tumefiaciens* gv2260 (PROGED::gPDS). Lane 1 shows the mutant PDS.28 g, the phenotype of which is shown in Fig. 3c. PCR was carried out with 34 cycles and fragments were separated on a 1.2% agarose gel. M, DNA ladder; D, non-transformed ‘Désirée’; PDS, PCR fragments amplified from intact genomic DNA; MlyI-PDS, PCR fragments amplified from *Mly*I-digested genomic DNA

**Fig. 3:**
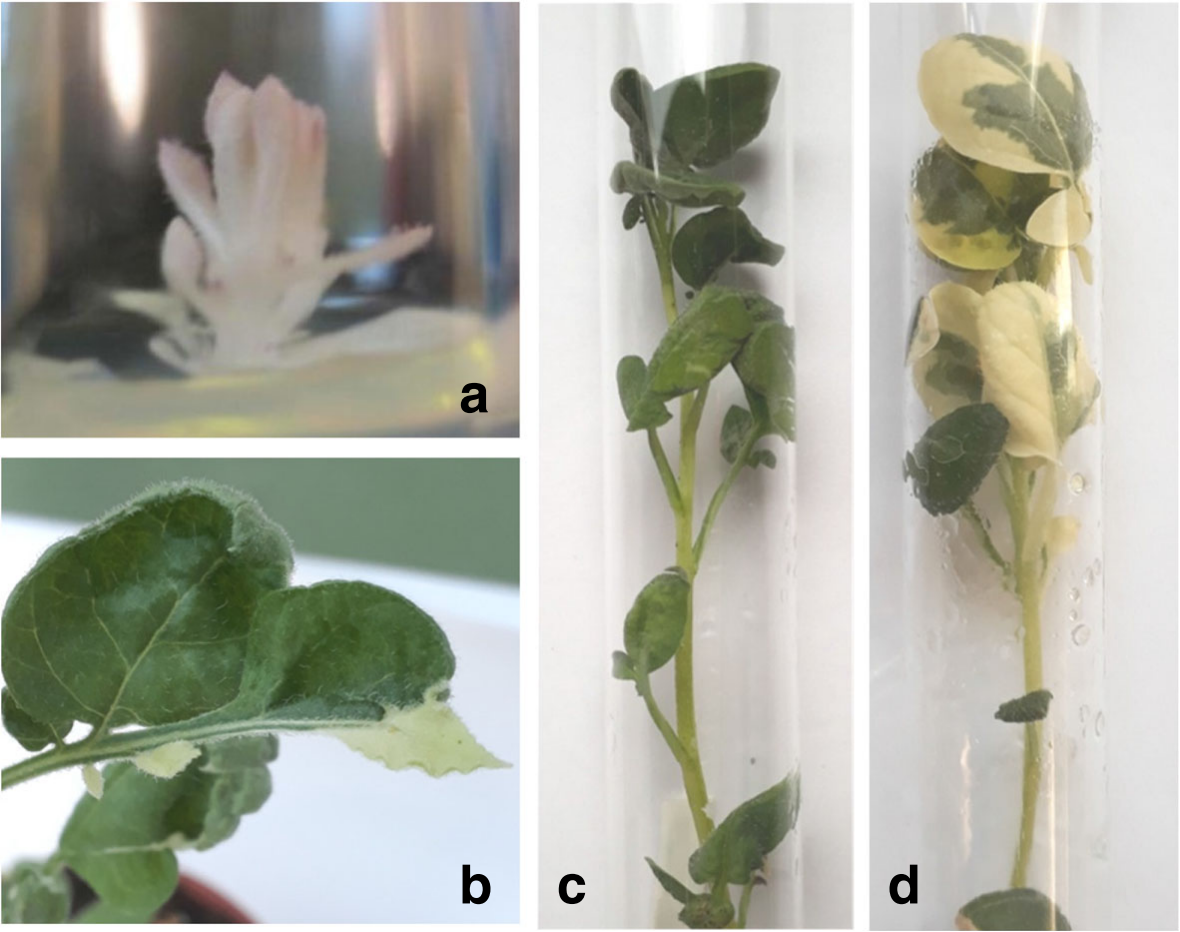
Phenotypes of *PDS* mutants. (**a**) PDS.3w - albino, (**b**) PDS.5 m -“tabby”, (**c**) PDS.28 g - green, indistinguishable from the wild-type, (**d**) PDS.10 m - “patchy” plant

In the first experiment, the efficiency of CP025 and PROGED vectors was compared using two *A. tumefaciens* strains, namely, gv2260 and LBA4404, in tuber transformation and with continuous Km selection for stable integration of gene-editing constructs. After regeneration and rooting, genomic DNA was isolated from the plants, and without and with digestion with *Mly*I, the 550-bp *PDS* fragment including the targeted region was PCR-amplified. The PCR fragments were separated on an agarose gel. In sum, 144 putative transgenic plants were obtained, 62 of which were analysed on gels. In addition to the original size, fragments smaller than 550 bp were detected in the majority of the samples (Fig. 2a), indicating large deletions in the targeted region. The frequency of mutation was notably high (47–88%). No substantial difference in the efficiency of CP025- and PROGED-mediated mutagenesis was found (Table 1). Two albino plants (one of them is shown in Fig. 3a) with large deletions were obtained. One chimaeric “tabby” plant characterised by white areas, especially at leaf edges (Fig. 3b), and a large deletion in the *PDS* region (Fig. 2, lane 9) was also found. All other plants were green and did not differ from the non-transformed ‘Désirée’ in phenotype (Fig. 3c).

**Table 1:** Efficiency of *PDS* mutagenesis with Km selection

Transformed organ	Strain	Vector	No. of regenerated plants	No. of tested plants^**a**^	No. of mutants	Efficiency^**b**^ (%)
Tuber	gv2260	CP025	36	16	11	69
		PROGED	24	8	7	88
	LBA4404	CP025	56	21	18	86
		PROGED	28	17	8	47
Leaf	gv2260	PROGED	24	14	9	64
	LBA4404		16	9	7	78

^a^ Plants for testing the presence of mutation in the *PDS* gene were selected randomly

^b^ Efficiency (%) of *PDS* mutagenesis was calculated as number of mutants detected by the restriction enzyme site loss method in PCR amplification of genomic DNA after digestion with *Mly*I / number of tested plants × 100

In the second experiment, PROGED with *gPDS* was used for leaf transformation by the two *Agrobacterium* strains and with continuous Km selection. The efficiency of leaf mutagenesis was similar to the efficiency of tuber mutagenesis. Based on PCR fragment detection in agarose gels, 16 *PDS* mutants were identified out of 40 regenerated plants (Table 1). The majority of these mutants also possessed a large deletion in the targeted region (Fig. 2b).

The presence and expression of *Cas9* was tested in seven *PDS* mutant plants by PCR and RT-PCR, respectively. *Cas9* was present in six plants. Low amounts of *Cas9* mRNA were detected in four plants (Additional file 3: Fig. S1). Except for albino plants, which were not rooted, the tested *PDS* mutants, including “tabby”, were maintained and propagated in vitro for more than a year. No obvious changes in phenotype were visible on the mutant lines.

### Testing the efficiency of PROGED-mediated gene editing with 2-week selection

The bi-functional translationally fused marker gene *codA-nptII* provides a positive selection for transformation events with Km in the media and negative selection for the presence/genomic insertion of transgenes with 5-fluorocytosine (5-FC) in the media. The negative selection is based on the enzymatic function of CodA, which converts the non-toxic 5-FC to cytotoxic 5-fluorouracil (5-FU) [17]. Earlier, we found that in the presence of recombinase, 3 weeks of positive selection followed by negative selection was optimal for the isolation of marker-free transgenic lines [15]. Since we removed the recombinase from the vector, a shorter, 2-week positive selection followed by negative selection was undertaken for *PDS* mutagenesis using the PROGED vector. Leaves and tubers were transformed in parallel with the *Agrobacterium* strains gv2260 and LBA4404. In total, 44 regenerated plants were obtained; however, based on the PCR test described above, none of them carried mutations in the targeted region (Additional file 1: Table S2).

### Testing the efficiency of PROGED-mediated gene editing with 3-day selection

The negative results of 2-week selection experiments indicated that the transgenes are in an integrated stage by this period of time. Therefore, a considerably shorter, 3-day Km selection was applied followed by lack of selection or with counter-selection of emerging calluses and regenerating shoots carrying integrated transgenes derived from PROGED::gPDS. In parallel, a transformation experiment without any selection for the transformation event was carried out as a control. Due to the short time or even lack of selection, hundreds of regenerated shoots appeared both from tuber and leaf transformation either by *Agrobacterium* gv2260 or LBA4404. We hypothesised that the majority of the regenerated plants are non-transformed. Therefore, genomic DNA was isolated from groups of plants to search for *PDS* mutants. Each group consisted of six plants. Seven groups were tested from each transformation with 3-day Km selection. Nineteen positive groups were identified. Individual testing of plants by PCR resulted in the identification of 22 *PDS* mutants appearing with a frequency of 2–10% with no substantial difference between counter-selected and non-counter-selected plant populations (Table 2). In contrast with the majority of the plants with integrated transgenes, none of the mutant plants had a deletion large enough to be visible on a gel in the PCR test. Two chimaeric plants with a green-white mosaic phenotype were obtained. One of them was indistinguishable from the “tabby” transgenic plant shown in Fig. 3b. The other one was “patchy” and possessed larger white areas than “tabby” (Fig. 3d). From the experiment with a lack of selection, 32 groups were tested. None of the groups harboured a *PDS* mutant. Thus, it was concluded that the chance to find a gene-edited mutant based on the restriction enzyme site loss method in a regenerated potato plant population without any selection is less than 0.5%.

**Table 2:** Efficiency of PROGED::gPDS mutagenesis with Km selection for 3 days

Transformed organ	Counter-selection	Strain	No. of groups with mutants^**a**^	Number of mutants	Efficiency (%)^**b**^
Tuber		gv2260	1	1	2
		LBA4404	2	3	7
	5-FC	gv2260	3	3	9
		LBA4404	2	2	5
Leaf		gv2260	2	3	9
		LBA4404	3	4	10
	5-FC	gv2260	2	2	5
		LBA4404	4	4	10

^a^ Seven groups of regenerated plants were tested from each transformation experiment. Each group consisted of six plants

^b^ Efficiency (%) of *PDS* mutagenesis was calculated as number of mutants detected by the restriction enzyme site loss method in PCR amplification of genomic DNA after digestion with *Mly*I / number of tested plants (7 groups × 6 plants = 42) × 100

Ten lines isolated after 3 days of selection, including the two chimaeras, were further investigated by PCR. Using primer pairs specific for the *Cas9-scfRNA* fragment, *Cas9* and *nptII,* it was concluded that the *PDS* mutations in these plants were generated from transient expression of *Cas9* and *gPDS,* as no PCR fragments derived from the above listed parts of PROGED::gPDS were detected on agarose gels (five lines are shown in Additional file 3: Fig. 2).

### Genotyping of *PDS* mutants at the DNA sequence level

The same region surrounding *gPDS*, which was sequenced from the non-transformed ‘Désirée’, was PCR-amplified, cloned into pGEM-T Easy and Sanger-sequenced from 15 *PDS* mutants originating from different experiments (Table 3). Commercial potato cultivars are tetraploids. The 10 sequenced ‘Désirée’ clones represented three characteristically different types of *PDS* genes, which may correspond to three different alleles of *PDS*. Arbitrarily choosing one of them as “wild-type”, a 2-bp AC deletion and a 16-bp insertion, respectively, were detected in the other two types (Additional file 2: Fig. S1). DNA sequence analysis of the clones derived from *PDS* mutants identified the fourth allele with a sequence alteration at a few bp upstream of the *gPDS* sequence (Additional file 2: Fig. S3,4,7,12).

**Table 3:** Phenotype and the detected mutations in the *PDS*-mutated potato plants

Tf. organ	Selection	Strain	Mutant	Pheno type	Genotype^*^			
					Allele 1	Allele 2 (−AC)	Allele 3 (+ 16 bp)	Allele 4 (variant)
Tuber	Km	gv2260	PDS.12 g	green	Δ165 (2) Δ5 (2)		Δ165 (1)	
			PDS.5 m	mosaic (tabby)	Δ1 (1) wt (2)		wt (1)	Δ165 (7)
			PDS.3w	white				Δ146 (1) Δ166 (2)
			PDS.11w	white	Δ104 (2)			
		LBA4404	PDS.28 g	green	Δ35 + T/A (5) wt (1)			
			PDS.34 g	green		wt (1)		Δ165 (4)
Tuber	3d-Km	gv2260	PDS.73 g	green	wt (3)	wt (2)	Δ1 (1)	
	3d-Km /5-FC		PDS.65 g	green	Δ1 (1) wt (3)	wt (1)		
	3d-Km	LBA4404	PDS.48 g	green	Δ2 + T/A (1) wt (1)	wt (2)		
			PDS.9 m	mosaic (tabby)	Δ3 (1)	Δ1 (3)	wt (3)	
	3d-Km /5-FC		PDS.10 m	mosaic (patchy)	wt (3)			Δ117 (1)
Leaf	3d-Km	gv2260	PDS.10 g	green	Δ1 + Δ3 (1) wt (2)	wt (1)		
	3d-Km	LBA4404	PDS.1 g	green	Δ2 (1) wt (6)			
			PDS.33 g	green	Δ2 + G/A (1) wt (8)			
	3d-Km /5-FC		PDS.5 g	green	wt (3)	Δ2 + T/C (1) wt (1)		

^*^Δ numbers indicate the size of the deletions in bp. Numbers in parenthesis specify the number of clones with identical sequence. /, base pair changes; wt, wild-type

The DNA sequence analysis supported the result of the PCR test because large deletions extending from 35 to 166 bp were detected in mutants obtained by continuous Km selection (Table 3 and Additional file 2: Fig. 2–7), while with temporary Km selection, deletions of only a few bp and 1-bp substitutions in three mutants were obtained (Table 3 and Additional file 2: Fig. 8–16). The mutations were extended to all four alleles; however, biallelic mutations were found only in three mutants (Table 3 and Additional file 2: Fig. S2,3,11). Wild-type and mutant sequences or two different mutations in the same allele were detected in eight out of ten green plants (Table 3 and Additional file 2: Fig. S2,6,9,10,13,14,15,16) indicating that not only “tabby” and “patchy”, but several green plants were also chimaeras. Due to the limited number of sequenced clones (Table 3), the presence of additional mutations, especially in the albino and chimaeric plants with white areas, is highly probable.

## Discussion

Producing transgene-free targeted mutants from vegetatively propagated, highly heterozygous and self-incompatible commercial potato cultivars is a challenging task. In this study, we tested the possibility of using positive/negative selection for mutant enrichment in a regenerating plant population obtained after *Agrobacterium*-mediated gene editing via Cas9 and sgRNA. The *PDS* gene was targeted because of the easily recognisable albino phenotype of null mutants. Since 100% identity between the DNA sequences of the 20-bp oligo used successfully for gene editing in tomato [16] and the corresponding region of the potato cultivar ‘Désirée’ was detected, we used this oligo for mutagenesis in the potato cultivar ‘Désirée’. Since the oligo carried a *Mly*I recognition site, the restriction enzyme site loss method was used to easily detect mutations in the targeted region by PCR. Nevertheless, it has been shown that Cas9 preferentially cleaves the targeted region and generates blunt-ended double-strand break (DSB) 3 bp upstream of PAM motif [18]. Thus, in our experiments, mutants with small indels not extending to the *Mly*I site located 14 bp upstream of PAM motif (Fig. 1) were lost.

A novel vector suitable for gene editing with positive/negative selection was constructed. It was demonstrated that using only the positive selection function of the new vector PROGED carrying only the right border (*RB*) sequence, the new vector is as efficient in gene-editing as the traditional vector pCAMBIA with *RB* and left border (*LB*) sequences. With continuous selection on Km, the frequency of mutations detected by the restriction enzyme site loss method varied between 47 and 88%. Similarly, high efficiencies were detected with *Agrobacterium*-mediated gene editing with stable transgene integration for the *ALS* gene in potato [6, 7, 14] or for *PDS* in other plant species: tomato, poplar*,* apple, rice, watermelon, cassava, grape, banana, coffee, pear, chicory, strawberry and cabbage [16, 19–30].

Using 2-week positive selection continued with negative selection, no transgene-free mutant plants could be isolated. The use of 3-day positive selection continued with negative selection generated *PDS* mutants with a frequency of 5–10%. This frequency, however, did not differ significantly from the efficiency of 3-day positive selection without subsequent negative selection, which varied from 2 to 10%, while without positive selection no mutants detectable by the restriction enzyme site loss method appeared. Using tobacco as a model plant and an intron-containing *GUS* gene as a marker, Chen et al. [13] also observed that transient expression of T-DNA genes in inoculated leaf discs peaked 3–4 days after *Agrobacterium* infection in the absence of Km selection. While in the case of tobacco, high-throughput mutant screening protocol had to be used for the identification of mutants [13], our results indicated that the *Agrobacterium*-mediated transformation and gene-editing by the CRISPR/Cas9 system is sufficiently efficient in potato that mutants can be obtained using a simple PCR screen of plants regenerated after 3 days of positive selection. This type of selection can be performed with any traditional binary vectors.

Stable integration of *Cas9* and *gPDS* generated large deletions extending up to 166 bp in the *PDS* gene. Such large deletions are rarely detected in other plant species. However, this phenomenon is not without examples, as large deletions were identified in the *Gn1a* gene of rice and the *ALARP* gene of cotton [31, 32]. Deletions larger than 100 bp are generally found only after mutagenesis with double or multiple sgRNA constructs [30, 33–38]. In potato, a deletion larger than 100 bp was detected in one out of nine mutants obtained in the Pi transport-related *PHO1* gene [39]. Makhotenko et al. [40] reported two potato mutants with deletions larger than 60 bp in the *COILIN* gene implicated in virus resistance, which were generated from apical meristem cells either by bioballistics with gold microparticles or infiltration with chitosan microparticles decorated with the Cas9/sgRNA RNP complex. Earlier publications with single sgRNA and transgene integration reported a maximum of 18 bp in the *IAA* gene and 17 bp in the *ALS* gene of potatoes [4, 6].

In contrast with stable integration, transient expression of *Cas9* and *gPDS* resulted in small deletions and with 1-bp substitutions in certain lines. The only exception was the line with large white patches harbouring a 117-bp deletion. Since the same construct was used for both types of experiments, we suppose that the difference is based on the different amounts of Cas9 and sgRNA produced in cells. While in the case of stable integration, both elements are produced continuously in large amounts upon regeneration, the transient expression provides only low amounts of components for a short period of time and opens the door to the DNA repair mechanism.

The targeted mutagenesis of the *PDS* gene in different plant species highlighted the general problem of chimaerism of *Agrobacterium*-mediated transgenesis. Green-white mosaic plants were isolated after *PDS* mutagenesis in tomato, rice, watermelon, cassava, grape, banana, apple, pear, strawberry and cabbage [16, 20–25, 27, 29, 30]. Mutation types and inheritance patterns in later generations were studied in tomato and rice [16, 21]. Out of tomato T_0_ plants, 64% had chimaeric mutations, in which at least two mutation types occurred in each, including deletions, insertions and combined mutations. In chimaeras, new mutation types were observed due to the existence of wild-type copies. Segregation patterns of the chimaeras were not predictable, and a number of new mutants were found in the T_1_ lines [16]. Unpredicted segregations, with more mutants than theoretically expected, were also frequently found in rice T_1_ plants, indicating that inherited *Cas9*s were still active in later generations and could induce new mutations in the progeny [21]. Although *Cas9* was also present in our *PDS* mutants obtained by continuous Km selection, the white areas did not grow on “tabby” plants, and no white areas appeared on green plants even after a year of vegetative propagation. Low amounts of *Cas9* mRNA could be detected in these lines by RT-PCR. Nevertheless, as we do not know the level of mosaicism, we cannot reach conclusions regarding the level of *Cas9* expression in those cells that harbour *Cas9*. The frequency of chimaeric mutations also cannot be calculated from our experiments because plants carrying mono-, bi-, or triallelic mutations in the *PDS* gene may be as green as wild-type plants and can be either chimaeric or non-chimaeric. Faize et al. [41] found that chimaeras are a highly frequent phenomenon observed after regenerating tobacco and apricot transgenic plants and suggested using real-time PCR quantification of the transgene in comparison to a housekeeping gene during sub-cultivation periods or in different organs if antibiotic selection is used. Chimaeras can be dissociated by iterative regeneration. This method is, however, a time-consuming approach with unpredictable outcomes. Crossing out the mutations from our *PDS* mutant plants would be necessary to get information on the frequency of chimaeras in *PDS*-edited population of potato generated by *Agrobacterium*-mediated transformation.

## Conclusions

This work shows that *Agrobacterium*-mediated gene editing in potatoes is an efficient method either by tuber or leaf transformation and using either *A. tumefaciens* strain gv2260 or LBA4404. Large deletions can be generated by continuous positive selection for stable transgene integration, while small deletions and nucleotide substitutions are derived from the transient expression of the Cas9 system. Stable integration of *Cas9* and *sgRNA* can generate mutations in all four copies of a gene in the tetraploid genome of potato, as demonstrated by the appearance of albino plants in the *PDS* mutant population. Nevertheless, irrespective of the selection method, chimaeras appear in the mutant population. Despite the presence of *Cas9* and *sgRNA* in the genome, the T_0_ plants propagated from stem segments had a stable phenotype, suggesting that the expression of *Cas9* and *sgRNA* is silenced or that the DNA repair system is highly active during the vegetative growth phase in potato. Gene function studies in potatoes have always been performed on the T_0_ generation of transgenic plants. Since the phenotype of the gene-edited transgenic lines propagated in vitro is stable, these plants are suitable for gene-function studies.

## Methods

### Vector constructions

The 20-bp oligo defined by [16] with *Aar*I sites was synthesised by Integrated DNA Technologies (IDT, Coralville, IA, USA) and inserted between the two *Aar*I sites of CP025 [4] as annealed oligonucleotides. The high-fidelity Phusion DNA polymerase (Thermo Fisher Scientific, Waltham, MA, USA) was used to amplify the *Cas9-gPDS* construct with the primers AscI35SFW and PacIScfRNAR (Additional file 1: Table S1) following the manufacturer’s instructions. The generated PCR fragment was digested with *Asc*I and *Pac*I and inserted between the corresponding sites of PROGMO [15], thereby replacing the recombinase in the vector and resulting in PROGED::gPDS (Fig. 1).

### Plant growth conditions and transformation

Virus-free and sterile shoots of the commercial potato (*Solanum tuberosum* L.) cultivar ‘Désirée’ were obtained from Fritz Lange KG (Bad Schwartau, Germany), cultivated under axenic conditions for tissue culture at the Max Planck Institute of Molecular Plant Physiology (Golm, Germany) [42] and transferred to our laboratory, where the plants were propagated in vitro from stem segments in MS medium (w/v) [43] without vitamins containing 2% (w/v) sucrose and solidified with 0.8% agar (rooting medium; RM) in tubes closed with paper plugs. For tuber transformation, the tops of the plants were transferred into 500-ml Erlenmeyer flasks (2 plants/flask) containing 160 ml RM and incubated in culture room at 24 °C under a light regime of 16 h light at 75 μmol m^− 2^ s^− 1^ intensity and 8 h of darkness. Tubers appeared on plants 4 months after planting. Tubers with a diameter of approximately 1 cm were cut to fine slices. The slices were co-cultured with the *Agrobacterium tumefaciens* strains gv2260 [44] or LBA4404 [45] carrying a recombinant plasmid for 2 days in the dark at 24 °C. Shoots were regenerated from tuber slices as described by [46] in 500-ml Erlenmeyer flasks closed with paper plugs in the presence of 500 mg l^− 1^ cefotaxime (Cf) to eliminate the *Agrobacterium* from the culture. The medium was supplemented with 50 mg l^− 1^ Km and/or 150 mg l^− 1^ 5-FC, depending on the experimental design. Regenerated shoots were excised and placed either into paper-plugged tubes or Phytatrays (Sigma, St. Louis, MI, USA) containing RM medium for rooting. The RM medium was supplemented with Cf or Cf + Km when regenerated shoots were derived from continuous Km selection*.*

For leaf transformation, the potato cultivar ‘Désirée’ was propagated in vitro in 500-ml jars in MS medium containing 2% (w/v) sucrose and solidified with 0.8% agar (5 plants/jars). Transformation and regeneration of shoots from leaves of 1-month-old plants was carried out according to [42]. The *Agrobacterium* strains, Km and 5-FC concentrations used were the same as for tuber transformation, but the concentration of Cf was decreased to 250 mg l^− 1^.

### Molecular biology techniques

For basic techniques, e.g., plasmid DNA isolation, digestion with restriction enzymes, transformation of *Escherichia coli*, agarose gel electrophoreses, PCR, etc. instructions of [47] were followed. Genomic DNA from potato was isolated according to [48], while RNA from leaves of in vitro-grown plants was purified by the method of [49]. The Maxima H Minus First Strand cDNA Synthsis Kit with ds DNAse (Thermo Fisher Scientific, Walthman, MA, USA) was used to convert mRNA to cDNA.

The primer pair PDS Fw and PDS R (Additional file 1: Table 1) were used for detection of mutations in *PDS* gene by PCR. Two hundred ng of potato genomic DNA was used as a template without digestion and after digestion with *Mly*I. The *Mly*I-digested DNA was precipitated by ethanol and sodium acetate, washed with 70% ethanol, dried and dissolved in distilled water [47] before using it for PCR.

Sanger-sequencing was performed at the company BIOMI (Gödöllő, Hungary) and analysed by NCBI blast (https://blast.ncbi.nlm.nih.gov/Blast.cgi) and the multiple alignment tool Clustal Omega (www.ebi.ac.uk/Tools/msa/clustalo).

## Supplementary information

**Additional file 1 Table S1.** Primer sequences; **Table S2.** Efficiency of *PDS* mutagenesis with Km selection for 2 weeks followed by counter-selection with 5-FC.

**Additional file 2 Figure S1.** Multiple sequence alignment of *PDS* fragments cloned from the potato cultivar ’Désirée’. **Figure S2.** PDS.12g. **Figure S3.** PDS.5m. **Figure S4.** PDS.3w. **Figure S5.** PDS.11w. **Figure S6.** PDS.28g. **Figure S7.** PDS.34g. **Figure S8.** PDS.73g. **Figure S9.** PDS.65g. **Figure S10.** PDS.48g. **Figure S11.** PDS.9m. **Figure S12.** PDS.10m. **Figure S13.** PDS.10g. **Figure S14.** PDS.1g. **Figure S15.** PDS.33g. **Figure S16.** PDS.5g.

**Additional file 3: Figure S1.** Detection and expression of *Cas9* in *PDS* mutants obtained after continuous Km selection. **Figure S2.** Detection of the lack of transgenes in *PDS* mutants obtained after 3 days of Km selection.

## Data Availability

All data generated or analysed during this study are included in this published article and its supplementary information files.

## Abbreviations

ALS: Acetolactate synthase
Cf: Cefotaxime
5-FC: 5-fluorocytosine
IAA: Indole acetic acid
Km: Kanamycin
PDS: Phytoene desaturase
