# Supplementary material for: Generation of transgene-free PDS mutants in potato by Agrobacterium-mediated transformation
Source: BMC Biotechnol. 2020 May 12;20:25. doi: 10.1186/s12896-020-00621-2 (PMC7216596; doi:10.1186/s12896-020-00621-2)
Supplement: Supplementary file 1 — Additional file 1 Table S1. Primer sequences; Table S2. Efficiency of PDS mutagenesis with Km selection for 2 weeks followed by counter-selection with 5-FC. [file 12896_2020_621_MOESM1_ESM.docx]

**Table S1**

**Primer sequences**

| **Name** | **Sequence (5’→3’)** |
| --- | --- |
| StgPDSFw | TCGGGGACTCTTGCCAGCAATGCT |
| StgPDSR | AAACAGCATTGCTGGCAAGAGTCC |
| PDS Fw | TTTCCCCGAAGCTTTACCCG |
| PDS R | ATCTGTCACCCTATCCGGCA |
| AscI35SFW | AACAGGCGCGCCATGATTACGAATTGGGTA |
| PacIScfRNAR | ACTGATTAATTAAACAAAAAAAGCACC |
| Cas9-59 Fw | CGGCGATGGACAAGAAGTAT |
| Cas9-1133 Fw | ACGGCTACGCTGGTTATATTG |
| Cas9-1462 R | GATGGTTTCCTCGCTCTTTCT |
| Cas9-1594 Fw | CATTCCCTCCTGTACGAGTATTT |
| Cas9-2019 R | CCATCCGGTGTATCTTCTC |
| npt II PROGMO Fw | TGGGCACAACAGACAATCGGCTGC |
| nptII PROGMO R | TGCGAATCGGGAGCGGCGATACCG |
| Actin Fw | TGGACTCTGGTGATGGTGTG |
| Actin R | GGTTTCAAGTTCCTGCTCGT |

Restriction enzyme recognition sites are underlined.

**Table S2**

**Efficiency of *PDS* mutagenesis with Km selection for 2 weeks followed by counter-selection with 5-FC**

|  | **Strain** | **Vector** | **No. of regenerated plants** | **No. of tested plants** | **No. of mutants** |
| --- | --- | --- | --- | --- | --- |
| Tuber | gv2260 | PROGED | 1 | 1 | 0 |
|  | LBA4404 |  | 17 | 17 | 0 |
| Leaf | gv2260 |  | 0 | - | - |
|  | LBA4404 |  | 26 | 21 | 0 |
