# Supplementary material for: Generation of transgene-free PDS mutants in potato by Agrobacterium-mediated transformation
Source: BMC Biotechnol. 2020 May 12;20:25. doi: 10.1186/s12896-020-00621-2 (PMC7216596; doi:10.1186/s12896-020-00621-2)
Supplement: Supplementary file 2 — Additional file 2 Figure S1. Multiple sequence alignment of PDS fragments cloned from the potato cultivar ’Désirée’. Figure S2. PDS.12g. Figure S3. PDS.5m. Figure S4. PDS.3w. Figure S5. PDS.11w. Figure S6. PDS.28g. Figure S7. PDS.34g. Figure S8. PDS.73g. Figure S9. PDS.65g. Figure S10. PDS.48g. Figure S11. PDS.9m. Figure S12. PDS.10m. Figure S13. PDS.10g. Figure S14. PDS.1g. Figure S15. PDS.33g. Figure S16. PDS.5g. [file 12896_2020_621_MOESM2_ESM.docx]

**Désirée**

D19 TTTCCCCGAAGCTTTACCCGCTCCTTTAAATGGTGAGCACATCATGAATAAATTTAGCCC 60

D1 TTTCCCCGAAGCTTTACCCGCTCCTTTAAATGGTGAGCACATCATAAATAAT-------- 52

D3 TTTCCCCGAAGCTTTACCCGCTCCTTTAAATGGTGAGCACATCATAAATAAT-------- 52

D8 TTTCCCCGAAGCTTTACCCGCTCCTTTAAATGGTGAGCACATCATAAATAAT-------- 52

D11 TTTCCCCGAAGCTTTACCCGCTCCTTTAAATGGTGAGCACATCATAAATAAT-------- 52

D7 TTTCCCCGAAGCTTTACCCGCTCCTTTAAATGGTGAGCACATCATAAATAAT-------- 52

D13 TTTCCCCGAAGCTTTACCCGCTCCTTTAAATGGTGAGCACATCATAAATAAT-------- 52

D2 TTTCCCCGAAGCTTTACCCGCTCCTTTAAATGGTGAGCACATCATAAATAAT-------- 52

D22 TTTCCCCGAAGCTTTACCCGCTCCTTTAAATGGTGAGCACATCATAAATAAT-------- 52

D9 TTTCCCCGAAGCTTTACCCGCTCCTTTAAATGGTGAGCACATCATAAATAAT-------- 52

********************************************* *****

D19 TCTGTAATCCTGTTGTCAAACTTCCCTAATAAGTTATTAAATTGATTATTCAATTACACC 120

D1 --------CATGTTGTCAA-ACTTCCCTAAAAGTTATTAAATTGATTATTCAATTACACC 103

D3 --------CATGTTGTCAA-ACTTCCCTAAAAGTTATTAAATTGATTATTCAATTACACC 103

D8 --------CATGTTGTCAA-ACTTCCCTAAAAGTTATTAAATTGATTATTCAATTACACC 103

D11 --------CATGTTGTCAA-ACTTCCCTAAAAGTTATTAAATTGATTATTCAATTACACC 103

D7 --------CATGTTGTCAA-NATTCCCTAAAAGTTATTAAATTGATTATTCAATTACACC 103

D13 --------CATGTTGCCAA-ACTTCCCTAAAAGTTATTAAATTGATTATTCAATTACACC 103

D2 --------CATGTTGTCAA-ACTTCCCTAAAAGTTATTAAATTGATTATTCAATTACACC 103

D22 --------CATGTTGTCAA-ACTTCCCTAAAAGTTATTAAATTGATTATTCAATTACACC 103

D9 --------CATGTTGTCAA-ACTTCCCTAAAAGTTATTAAATTGATTATTCAATTACACC 103

* ***** *** * ** * ******************************

D19 TTTGGGACTTTACTAACCTTAAAAGAGCATTAAGGTTCATTACTTCTTCATCGGACCTTT 180

D1 TATGGGACTTTACTAACCTTAAAAGAGCAGTAAGGTTCATTACTTCTTCATCGGACCTTT 163

D3 TATGGGACTTTACTAACCTTAAAAGAGCAGTAAGGTTCATTACTTTTTCATCGGACCTTT 163

D8 TATGGGACTTTACTAACCTTAAAGGAGCAGTAAGGTTCATTACTTCTTCATCGGACCTTT 163

D11 TATGGGACTTTACTAACCTTAAAAGAGCAGTAAGGTTCATTACTTCTTCATCGGACCTTT 163

D7 TATGGGACTTTACTAACCTTAAAAGAGCNNTANGGTTCATTACTTTTTCATCGGACCTTT 163

D13 TATGGGACTTTACTAACCTTAAAAGAGCAGTAAGGTTCATTACTTTTTCATCGGACCTTT 163

D2 TATGGGACTTTACTAACCTTAAAAGAGCAGTAAGGTTCATTACTTTTTCATCGGACCTTT 163

D22 TATGGGACTTTACTAACCTTAAAAGAGCAGTAAGGTTCATTACTTTTTCATCGGACCTTT 163

D9 TATGGGACTTTACTAACCTTAAAAGAGCAGTAAGGTTCATTACTTTTTCATCGGACCTTT 163

* ********************* **** ** ************ **************

D19 TGTGTGCAGCTAAAGTGTTAACTGCTTTGATATAATTTGCAGGAGTTTTGGCCATCCTAA 240

D1 TGTGTGCAGCTAAAGTGTTAACTGCTTTGATATAATTTGCAGGAGTTTTGGCCATCCTAA 223

D3 TGTGTGCAGCTAAAGTGTTAACTGCTTTGATATAATTTGCGGGAGTTTTGGCCATCCTAA 223

D8 TGTGTGCAGCTAAAGTGTTAACTGCTTTGATATAATTTGCAGGAGTTTTGGCCATCCTAA 223

D11 TGTGTGCAGCTAAAGTGTTAACTGCTTTGATATAATTTGCAGGAGTTTTGGCCATCCTAA 223

D7 TGTGTGCAGCTAAAGTGTTAACTGCTTTGATATAATTTGCAGGAGTTTTGGCCATCCTAA 223

D13 TGTGTGCAGCTAAAGTGTTAACTGCTTTGATATAATTTGCAGGAGTTTTGGCCATCCTAA 223

D2 TGTGTGCAGCTAAAGTGTTAACTGCTTTGATATAATTTGCAGGAGTTTTGGCCATCCTAA 223

D22 TGTGTGCAGCTAAAGTGTTAACTGCTTTGATATAATTTGCAGGAGTTTTGGCCATCCTAA 223

D9 TGTGTGCAGCTAAAGTGTTAACTGCTTTGATATAATTTGCAGGAGTTTTGGCCATCCTAA 223

**************************************** *******************

D19 AGAACAATGAAATGCTTACATGGCCAGAGAAAGTCAAATTTGCAATTGGACTCTTGCCAG 300

D1 AGAACAATGAAATGCTTACATGGCCAGAGAAAGTCAAATTTGCAATTGGACTCTTGCCAG 283

D3 AGAACAATGAAATGCTTACATGGCCAGAGAAAGTCAAATTTGCAATTGGACTCTTGCCAG 283

D8 AGAACAATGAAATGCTTACATGGCCAGAGAAAGTCAAATTTGCAATTGGACTCTTGCCAG 283

D11 AGAACAATGAAATGCTTACATGGCCAGAGAAAGTCAAATTTGCAATTGGACTCTTGCCAG 283

D7 AGAACAATGAAATGCTTACATGGCCAGAGAAAGTCAAATTTGCAATTGGACTCTTGCCAG 283

D13 AGAACAATGAAATGCTTACATGGCCAGAGAAAGTCAAATTTGCAATTGGACTCTTGCCAG 283

D2 AGAACAATGAAATGCTTACATGGCCAGAGAAAGTCAAATTTGCAATTGGACTCTTGCCAG 283

D22 AGAACAATGAAATGCTTACATGGCCAGAGAAAGTCAAATTTGCAATTGGACTCTTGCCAG 283

D9 AGAACAATGAAATGCTTACATGGCCAGAGAAAGTCAAATTTGCAATTGGACTCTTGCCAG 283

************************************************************

D19 CAATGCTTGGAGGGCAATCTTATGTTGAGGCTCAAGATGGGATAAGTGTCAAGGACTGGA 360

D1 CAATGCTTGGAGGGCAATCTTATGTTGGGGCTCAAGATGGGATAAGTGTCAAGGACTGGA 343

D3 CAATGCTTGGAGGGCAATCTTATGTTGAGGCTCAAGATGGGATAAGTGTCAAGGACTGGA 343

D8 CAATGCTTGGAGGGCAATCTTATGTTGAAGCTCAAGACGGGATAAGTGTTAAGGACTGGA 343

D11 CAATGCTTGGAGGGCAATCTTATGTTGAAGCTCAAGACGGGATAAGTGTTAAGGACTGGA 343

D7 CAATGCTTGGAGGGCAATCTTATGTTGAAGTTCAAGACGGGATAAGTGTTAAGGACTGGA 343

D13 CAATGCTTGGAGGGCAATCTTATGTTGAAGCTCAAGACGGGGTAAGTGTTAAGGACTGGA 343

D2 CAATGCTTGGAGGGCAATCTTATGTTGAAGCTCAAGACGGGATAAGTGTTAAGGACTGGA 343

D22 CAATGCTTGGAGGGCAATCTTATGTTGAAGCTCAAGACGGGATAAGTGTTAAGGACTGGA 343

D9 CAATGCTTGGAGGGCAATCTTATGTTGAAGCTCAAGACGGGATAAGTGTTAAGGACTGGA 343

*************************** * ****** *** ******* **********

D19 TGAGAAAGCAAGTGCGTAATCAGTTATGTTACTTTTTAAGCATAAACCTGGCTGTATAGT 420

D1 TGAGAAAGCAAGTGCGTAAACAGTTATGTTACTTTTTAAGCATAAACCTGGCTGTATAGT 403

D3 TGAGAAAGCAAGTGCGTAATCAGTTATGTTACTTTTTAAGCATAAACCTGGCTGTATAGT 403

D8 TGAGAAAGCAAGTGCGTAATTAGTTATGTTACTTTTTAAGCATAAACCTGGCTGTATTGT 403

D11 TGAGAAAGCAAGTGCGTAATTAGTTATGTTACTTTTTAAGCATAAACCTGGCTGTATTGT 403

D7 TGAGAAAGCAAGTGCGTAATTAGTTATGTTACTTTTTAAGCATAAACCTGGCTGTATAGT 403

D13 TGAGAAAGCAAGTGCGTAATTAGTTATGTTACTTTTTAAGCATAAACCTGGCTGTATAGT 403

D2 TGAGAAAGCAAGTGCGTAATTAGTTATGTTACTTTTTAAGCATAAACCTGGCTGTATAGT 403

D22 TGAGAAAGCAAGTGCGTAATTAGTTATGTTACTTTTTAAGCATAAACCTGGCTGTATAGT 403

D9 TGAGAAAGCAAGTGCGTAATTAGTTATGTTACTTTTTAAACATAAACCTGGCTGTATAGT 403

******************* ****************** ***************** **

D19 TCACAGTTCACACACAAATTGTTCATTTGAGGTGTTAGATTGTCTACCTGCCTTTCTTTT 480

D1 TCACAGTTCACACACAAATTGTTCATTTGAGGTGTTAGATTGTCTACCTGCCTTTCTTTT 463

D3 TCACAGTTCACACACAAATTGTTCATTTGAGGTGTTAGATTGTCTACCTGCCTTTCTTTT 463

D8 TCACAGTTCACACACAAATTGTTCGTTTGAGGTGTTAGATTGTCTACCTGCCTTTCTTTT 463

D11 TCACAGTTCACACACAAATTGTTCATTTGAGGTGTTAAATTGTCTACCTGCCTTTCTTTT 463

D7 TCACAGTTCACAC--ACATTGTTCATTTGAGGTGTTAGATTGTCTACCTGCCTTTCTTTT 461

D13 TCACAGTTCACAC--ACATTGTTCATTTGAGGTGTTAGATTGTCTACCTGCCTTTCTTTT 461

D2 TCACAGTTCACAC--ACATTGTTCATTTGAGGTGTTAGATTGTCTACCTGCCTTTCTTTT 461

D22 TCACAGTTCACAC--ACATTGTTCATTTGAGGTGTTAGATTGTCTACCTGCCTTTCTTTT 461

D9 TCACAGTTCACAC--ACATTGTTCATTTGAGGTGTTAGATTGTCTACCTGCCTTTCTTTT 461

************* * ******* ************ **********************

D19 GCTAACAGCATACTTCTAGCAAATCTCAGCAGCAGCAATTTGTTTTGCAGGGTGTGCCGG 540

D1 GCTAACAGCATACTTCTAGCAAATCTCAGCAGCAGCAATTTGTTTTGCAGGGTGTGCCGG 523

D3 GCTAACAGCATACTTCTAGCAAATCTCAGCAGCAGCAATTTGTTTTGCAGGGTGTGCCGG 523

D8 GCTAGCCGCATACATCTAGCAAATCTTAGCCGCAGCAATTTGTTTTGCAGGGTGTGCCGG 523

D11 GCTAGCCGCATACATCTAGCAAATCTTAGCCGCAGCAATTTGTTTTGCAGGGTGTGCCGG 523

D7 GCTAGCAGCATACATCTAGCAAATCTTAGCCGCAGCAATTTGTTTTGCAGGGTGTGCCGG 521

D13 GCTAGCAGCATACATCTAGCAAATCTTAGCCGCAGCAATTTGTTTTGCAGGGTGTGCCGG 521

D2 GCTAGCAGCATACATCTAGCAAATCTTAGCCGCAGCAATTTGTTTTGCAGGGTGTGCCGG 521

D22 GCTAGCAGCATACATCTAGCAAATCTTAGCCGCAGCAATTTGTTTTGCAGGGTGTGCCGG 521

D9 GCTAGCAGCATACATCTAGCAAATCTTAGCCGCAGCAATTTGTTTTGCAGGGTGTGCCGG 521

**** * ****** ************ *** *****************************

D19 ATAGGGTGACAGAT 554

D1 ATAGGGTGACAGAT 537

D3 ATAGGGTGACAGAT 537

D8 ATAGGGTGACAGAT 537

D11 ATAGGGTGACAGAT 537

D7 ATAGGGTGACAGAT 535

D13 ATAGGGTGACAGAT 535

D2 ATAGGGTGACAGAT 535

D22 ATAGGGTGACAGAT 535

D9 ATAGGGTGACAGAT 535

**************

**Fig. S1** Multiple sequence alignment using Clustal Omega of *PDS* fragments cloned from the potato cultivar ’Désirée’. The 20-bp region corresponding to *gPDS* is in red and blue colours. The *Mly*I recognition site is in blue. Allelic differences are highlighted in green.

**PDS.12g GREEN plant**

28.12_4 -TTCCCCGAAGCTTTACCCGCTCCTTTAAATGGTGAGCACATCATGAATAAATTTAGCCC 59

WT TTCCCCGAAGCTTTACCCGCTCCTTTAAATGGTGAGCACATCATAAATAATC------- 53

28.12_5 -TTCCCCGAAGCTTTACCCGCTCCTTTAAATGGTGAGCACATCATAAATAATC------- 52

28.12_6 -TTCCCCGAAGCTTTACCCGCTCCTTTAAATGGTGAGCACATCATAAATAATC------- 52

28.12_1 -TTCCCCGAAGCTTTACCCGCTCCTTTAAATGGTGAGCACATCATAAATAATC------- 52

28.12_2 -TTCCCCGAAGCTTTACCCGCTCCTTTAAATGGTGAGCACATCATAAATAATC------- 52

******************************************** *****

28.12_4 TCTGTAATCCTGTTGTCAAACTTCCCTAATAAGTTATTAAATTGATTATTCAATTACACC 119

WT ---------ATGTTGTCAA-ACTTCCCTAAAAGTTATTAAATTGATTATTCAATTACACC 103

28.12_5 ---------ATGTTGTCAA-ACTTCCCTAAAAGTTATTAAATTGATTATTCAATTACACC 102

28.12_6 ---------ATGTTGTCAA-ACTTCCCTAAAAGTTATTAAATTGATTATTCAATTACACC 102

28.12_1 ---------ATGTTGTCAA-ACTTCCCTAAAAGTTATTAAATTGATTATTCAATTACACC 102

28.12_2 ---------ATGTTGTCAA-ACTTCCCTAAAAGTTATTAAATTGATTATTCAATTACACC 102

********* * ** * ******************************

28.12_4 TATGGGACTTTACTAACCTTAAAAGAGCAGTAAGGTTCATTACCTCTTCATCGGACCTTT 179

WT TATGGGACTTTACTAACCTTAAAAGAGCAGTAAGGTTCATTACTTCTTCATCGGACCTTT 163

28.12_5 TATGGGACTTTACTAACCTTAAAAGAGCAGTAAGGTTCATTACTTCTTCATCGGACCTTT 162

28.12_6 TATGGGACTTTACTAACCTTAAAAGAGCAGTAAGGTTCATTACTTCTTCATCGGACCTTT 162

28.12_1 TATGGGACTTTACTAACCTTAAAAGAGCAGTAAGGTTCATTACTTCTTCATCGGACCTTT 162

28.12_2 TATGGGACTTTACTAACCTTAAAAGAGCAGTAAGGTTCATTACTTCTTCATCGGACCTTT 162

******************************************* ****************

28.12_4 TGTGTGCAGCTAAAGTGTTAACTGCTTTGA------------------------------ 209

WT TGTGTGCAGCTAAAGTGTTAACTGCTTTGATATAATTTGCAGGAGTTTTGGCCATCCTAA 223

28.12_5 TGTGTGCAGCTAAAGTGTTAACTGCTTTGATATAATTTGCAGGTTACTTTTTAAGCA--- 219

28.12_6 TGTGTGCAGCTAAAGTGTTAACTGCTTTGATATAATTTGCAGGTTACTTTTTAAGCA--- 219

28.12_1 TGTGTGCAGCTAAAGTGTTAACTGCTTTGATATAATTTGCAGGAGTTTTGGCCATCCTAA 222

28.12_2 TGTGTGCAGCTAAAGTGTTAACTGCTTTGATATAATTTGCAGGAGTTTTGGCCATCCTAA 222

******************************

28.12_4 ------------------------------------------------------------ 209

WT AGAACAATGAAATGCTTACATGGCCAGAGAAAGTCAAATTTGCAATTGGACTCTTGCCAG 283

28.12_5 ------------------------------------------------------------ 219

28.12_6 ------------------------------------------------------------ 219

28.12_1 AGAACAATGAAATGCTTACATGGCCAGAGAAAGTCAAATTTGCAATTGGACTCTTGCCAG 282

28.12_2 AGAACAATGAAATGCTTACATGGCCAGAGAAAGTCAAATTTGCAATTGGACTCTTGCCAG 282

28.12_4 ------------------------------------------------------------ 209

WT CAATGCTTGGAGGGCAATCTTATGTTGGGGCTCAAGATGGGATAAGTGTCAAGGACTGGA 343

28.12_5 ------------------------------------------------------------ 219

28.12_6 ------------------------------------------------------------ 219

28.12_1 -----CTTGGAGGGCAATCTTATGTTGAAGCTCAAGACGGGATAAGTGTTAAGGACCGGA 337

28.12_2 -----CTTGGAGGGCAATCTTATGTTGAAGCTCAAGACGGGATAAGTGTTAAGGACTGGA 337

28.12_4 ---------------TATAATTGGCAGGTTACTTTTTAAGCATAAACCTGGCTGTATTGT 254

WT TGAGAAAGCAAGTGCGTAAACAGTTATGTTACTTTTTAAGCATAAACCTGGCTGTATAGT 403

28.12_5 ------------------------------------------TAAACCTGGCTGTATTGT 237

28.12_6 ------------------------------------------TAAACCTGGCTGTATTGT 237

28.12_1 CGAGAAAGCAAGTGCGTAATTAGTTATGTTACTTTTTAAGCATAAACCTGGCTGTATTGT 397

28.12_2 TGAGAAAGCAAGTGCGTAATTAGTTATGTTACTTTTTAAGCATAAACCTGGCTGTATTGT 397

*************** **

28.12_4 TCACAGTTCACACACAAATTGTTCATTTGAGGTGTTAGATTGTCTACCTGCCTTTCTTTT 314

WT TCACAGTTCACACACAAATTGTTCATTTGAGGTGTTAGATTGTCTACCTGCCTTTCTTTT 463

28.12_5 TCACAGTTCACACACAAATTGTTCATTTGAGGTGTTGGATTGTCTACCTGCCTTTCTTTT 297

28.12_6 TCACAGTTCACACACAAATTGTTCATTTGAGGTGTTAGATTGTCTACCTGCCTTTCTTTT 297

28.12_1 TCACAGTTCACACACAAATTGTTCATTTGAGGTGTTAGATTGTCTACCTGCCTTTCTTTT 457

28.12_2 TCACAGTTCACACACAAATTGTTCATTTGAGGTGTTAGATTGTCTACCTGCCTTTCTTTT 457

************************************ ***********************

28.12_4 GCTAGCCGCATACATCTAGCAAATCTTAGCCGCAGCAATTTGTTTTGCAGGGTGTGCCGG 374

WT GCTAACAGCATACTTCTAGCAAATCTCAGCAGCAGCAATTTGTTTTGCAGGGTGTGCCGG 523

28.12_5 GCTAGCCGCATACATCTAGCAAATCTTAGCCGCAGCAATTTGTTTTGCAGGGTGTGCCGG 357

28.12_6 GCTAGCCGCATACATCTAGCAAATCTTAGCCGCAGCAATTTGTTTTGCAGGGTGTGCCGG 357

28.12_1 GCTAGCCGCATACATCTAGCAAATCTTAGCCGCAGCAATTTGTTTCGCAGGGTGTGCCGG 517

28.12_2 GCTAGCCGCATACATCTAGCAAATCTTAGCCGCAGCAATTTGTTTTGCAGGGTGTGCCGG 517

**** * ****** ************ *** ************** **************

28.12_4 ATAGGGTGACAGAT 388

WT ATAGGGTGACAGAT 537

28.12_5 ATAGGGTGACAGAT 371

28.12_6 ATAGGGTGACAGAT 371

28.12_1 ATAGGGTGACAGAT 531

28.12_2 ATAGGGTGACAGAT 531

**************

**Fig. S2** Multiple sequence alignment using Clustal Omega of *PDS* fragments cloned from the green mutant plant PDS.12g. WT, DNA sequence of the D1 clone isolated from non-transformed ‘Désirée’. The 20-bp region corresponding to the sgRNA is in red and blue colours. The *Mly*I recognition site is in blue. Differences compared to WT are highlighted in green.

**PDS.5m MOSAIC plant „tabby”**

5m.13 TTTCCCCGAAGCTTTACCCGCTCCTTTAAATGGTGAGCACATCATGAATAAATTTAGCCC 60

5m.19 TTTCCCCGAAGCTTTACCCGCTCCTTTAAATGGTGAGCACATCATAAATAATC------- 53

5m.18b TTTCCCCGAAGCTTTACCCGCTCCTTTAAATGGTGAGCACATCATAAATAATC------- 53

5m.17 TTTCCCCGAAGCTTTACCCGCTCCTTTAAATGGTGAGCACATCATAAATAATC------- 53

5m.1 TTTCCCCGAAGCTTTACCCGCTCCTTTAAATGGTGAGCACATCATAAATAATC------- 53

5m.16 TTTCCCCGAAGCTTTACCCGCTCCCTTAAATGGTGAGCACATCATAAATAATC------- 53

5m.3 TTTCCCCGAAGCTTTACCCGCTCCTTTAAATGGTGAGCACATCATAAATAATC------- 53

5m.18a TTTCCCCGAAGCTTTACCCGCTCCTTTAAATGGTGAGCACATCATAAATAATC------- 53

5m.2 TTTCCCCGAAGCTTTACCCGCTCCTTTAAATGGTGAGCACATCATAAATAATC------- 53

5m.9 TTTCCCCGAAGCTTTACCCGCTCCTTTAAATGGTGAGCACATCATAAATAATC------- 53

WT TTTCCCCGAAGCTTTACCCGCTCCTTTAAATGGTGAGCACATCATAAATAATC------- 53

5m.10 TTTCCCCGAAGCTTTACCCGCTCCTTTAAATGGTGAGCACATCATAAATAATC------- 53

************************ **************************

5m.13 TCTGTAATCCTGTTGTCAAACTTCCCTGATAAGTTATTAAATTGATTATTCAATTACACC 120

5m.19 ---------ATGTTGTCA-AACTTCCCTAAAAGTTATTAAATTGATTATTCAATTACACC 103

5m.18b ---------ATGTTGTCA-AACTTCCCTAAAAGTTATTAAATTGATTATTCAATTACACC 103

5m.17 ---------ATGTTGTCA-AACTTCCCTAAAAGTTATTAAATTGATTATTCAATTACACC 103

5m.1 ---------ATGTTGTCA-AACTTCCCTAAAAGTTATTAAATTGATTATTCAATTACACC 103

5m.16 ---------ATGTTGTCA-AACTTCCCTAAAAGTTATTAAATTGATTATTCAATTACACC 103

5m.3 ---------ATGTTGTCA-AACTTCCCTAAAAGTTATTAAATTGATTATTCAATTACACC 103

5m.18a ---------ATGTTGTCA-AACTTCCCTAAAAGTTATTAAATTGATTATTCAATTACACC 103

5m.2 ---------ATGTTGTCA-AACTTCCCTAAAAGTTATTAAATTGATTATTCAATTACACC 103

5m.9 ---------ATGTTGTCA-AACTTCCCTAAAAGTTATTAAATTGATTATTCAATTACACC 103

WT ---------ATGTTGTCA-AACTTCCCTAAAAGTTATTAAATTGATTATTCAATTACACC 103

5m.10 ---------ATGTTGTCA-AACTTCCCTAAAAGTTATTAAATTGATTATTCAATTACACC 103

******** * * ** * ******************************

5m.13 TTTGGGACTTTACTAACCTTAAAAGAGCATTAAGGTTCATTACTTCTTCATCGGACCTTT 180

5m.19 TATGGGACTTTACTAACCTTAAAAGAGCAGTAAGGTTCATTACTTCTTCATCGGACCTTT 163

5m.18b TATGGGACTTTACTAACCTTAAAAGAGCAGTAAGGTTCATTACCTCTTCATCGGACCTTT 163

5m.17 TATGGGACTTTACTAACCTTAAAAGAGCAGTAAGGTTCATTACTTCTTCATCGGACCTTT 163

5m.1 TATGGGACTTTACTAACCTTAAAAGAGCAGTAAGGTTCATTACTTCTTCATCGGACCTTT 163

5m.16 TATGGGACTTTACTAACCTTAAAAGAGCAGTAAGGTTCATTACTTCTTCATCGGACCTTT 163

5m.3 TATGGGACTTTACTAACCTTAAAAGAGCAGTAAGGTTCATTACTTCTTCATCGGACCTTT 163

5m.18a TATGGGACTTTACTAACCTTAAAAGAGCAGTAAGGTTCATTACTTCTTCATCGGACCTTT 163

5m.2 TATGGGACTTTACTAACCTTAAAAGAGCAGTAAGGTTCATTACTTCTTCATCGGACCTTT 163

5m.9 TATGGGACTTTACTAACCTTAAAAGAGCAGTAAGGTTCATTACTTCTTCATCGGACCTTT 163

WT TATGGGACTTTACTAACCTTAAAAGAGCAGTAAGGTTCATTACTTCTTCATCGGACCTTT 163

5m.10 TATGGGACTTTACTAACCTTAAAAGAGCAGTAAGGTTCATTACTTCTTCATCGGACCTTT 163

* *************************** ************* ****************

5m.13 TGTGTGCAGCTAAAGTGTTAACTGCTTTGATATAATTTGCAGGAGTTTTGGCCATCCTAA 240

5m.19 TGTGTGCAGCTAAAGTGTTAACTGCTTTGATATAATTTGCAGGAGTTTTGGCCATCCTAA 223

5m.18b TGTGTGCAGCTAAAGTGTTAACTGCTTTGATATAATTTGCAGGTTACTTTTTAAGCATAA 223

5m.17 TGTGTGCAGCTAAAGTGTTAACTGCTTTGAGATAATTTGCGGGTTACTTTTTAAGCATAA 223

5m.1 TGTGTGCAGCTAAAGTGTTAACTGCTTTGATATAATTTGCAGGTTACTTTTTAAGCATAA 223

5m.16 TGTGTGCAGCTAAAGTGTTAACTGCTTTGATATAATTTGCAGGTTACTTTTTAAGCATAA 223

5m.3 TGTGTGCAGCTAAAGTGTTAACTGCTTTGATATAATTTGCAGGTTACTTTTTAAGCATAA 223

5m.18a TGTGTGCAGCTAAAGTGTTAACTGCTTTGATATAATTTGCAGGTTACTTTTTAAGCATAA 223

5m.2 TGTGTGCAGCTAAAGTGTTAACTGCTTTGATATAATTTGCAGGTTACTTTTTAAGCATAA 223

5m.9 TGTGTGCAGCTAAAGTGTTAACTGCTTTGATATAATTTGCAGGAGTTTTGGCCATCCTAA 223

WT TGTGTGCAGCTAAAGTGTTAACTGCTTTGATATAATTTGCAGGAGTTTTGGCCATCCTAA 223

5m.10 TGTGTGCAGCTAAAGTGTTAACTGCTTTGATATAATTTGCAGGAGTTTTGGCCATCCTAA 223

****************************** ********* ** ** * * ***

5m.13 AGAACAATGAAATGCTTACATGGCCAGAGAAAGTCAAATTTGCAATTGGACTCTTGCCAG 300

5m.19 AGAACAATGAAATGCTTACATGGCCAGAGAAAGTCAAATTTGCAATTGGACTCTTGCCAG 283

5m.18b ACCTGGCTGTATTGC--------------------------------------------- 238

5m.17 ACCTGGCTGTATTGT--------------------------------------------- 238

5m.1 ACCTGGCTGTATTGT--------------------------------------------- 238

5m.16 ACCTGGCTGTATTGT--------------------------------------------- 238

5m.3 ACCTGGCTGTATTGT--------------------------------------------- 238

5m.18a ACCTGGCTGTATTGT--------------------------------------------- 238

5m.2 ACCTGGCTGTATTGT--------------------------------------------- 238

5m.9 AGAACAATGAAATGCTTACATGGCCAGAGAAAGTCAAATTTGCAATTGGACTCTTGCCAG 283

WT AGAACAATGAAATGCTTACATGGCCAGAGAAAGTCAAATTTGCAATTGGACTCTTGCCAG 283

5m.10 AGAACAATGAAATGCTTACATGGCCAGAGAAAGTCAAATTTGCAATTGGACTCTTGCCAG 283

* ** * **

5m.13 CAATGCTTGGAGGGCAATCTTATGTTGAGGCTCAAGATGGGATAAGTGTCAAGGACTGGA 360

5m.19 CAATC-TTGGAGGGCAATCTTATGTTGAAGCTCAAGACGGGATAAGTGTTAAGGACTGGA 342

5m.18b ------------------------------------------------------------ 238

5m.17 ------------------------------------------------------------ 238

5m.1 ------------------------------------------------------------ 238

5m.16 ------------------------------------------------------------ 238

5m.3 ------------------------------------------------------------ 238

5m.18a ------------------------------------------------------------ 238

5m.2 ------------------------------------------------------------ 238

5m.9 CAATGCTTGGAGGGCAATCTTATGTTGAGGCTCAAGATGGGATAAGTGTCAAGGACTGGA 343

WT CAATGCTTGGAGGGCAATCTTATGTTGGGGCTCAAGATGGGATAAGTGTCAAGGACTGGA 343

5m.10 CAATGCTTGGAGGGCAATCTTATGTTGAGGCTCAAGATGGGATAAGTGTCAAGGACTGGA 343

5m.13 TGAGAAAGCAAGTGCGTAATCAGTTATGTTACTTTTTAAGCATAAACCTGGCTGTATAGT 420

5m.19 TGAGAAAGCAAGTGCGTAATTAGTTATGTTACTTTTTAAGCATAAACCTGGCTGTATTGT 402

5m.18b ------------------------------------------------------------ 238

5m.17 ------------------------------------------------------------ 238

5m.1 ------------------------------------------------------------ 238

5m.16 ------------------------------------------------------------ 238

5m.3 ------------------------------------------------------------ 238

5m.18a ------------------------------------------------------------ 238

5m.2 ------------------------------------------------------------ 238

5m.9 TGAGAAAGCAAGTGCGTAATCAGTTATGTTACTTTTTAAGCATAAACCTGGCTGTATAGT 403

WT TGAGAAAGCAAGTGCGTAAACAGTTATGTTACTTTTTAAGCATAAACCTGGCTGTATAGT 403

5m.10 TGAGAAAGCAAGTGCGTAATCAGTTATGTTACTTTTTAAGCATAAACCTGGCTGTATAGT 403

5m.13 TCACAGTTCACACACAAATTGTTCATTTGAGGTGTTAGATTGTCTACCTGCCTTTCTTTT 480

5m.19 TCACAGTTCACACACAAATTGTTCATTTGAGGTGTTAGATTGTCTACCTGCCTTTCTTTC 462

5m.18b TCACAGTTCACGCACAAATTGTTCATTTGAGGTGTTAGATTGTCTACCTGCCCTTCTTTT 298

5m.17 TCACAGTTCACACACAAATTGTTCATTTGAGGTGTTAGATTGTCTACCTGCCTTTCTTTT 298

5m.1 TCACAGTTCACACACAAATTGTTCATTTGAGGTGTTAGATTGTCTACCTGCCTTTCTTTT 298

5m.16 TCACAGTTCACACACAAATTGTTCATTTGAGGTGTTAGATTGTCTACCTGCCTTTCTTTT 298

5m.3 TCACAGTTCACACACAAATTGTTCATTTGAGGTGTTAGATTGTCTACCTGCCTTTCTTTT 298

5m.18a TCACAGTTCACACACAAATTGTTCATTTGAGGTGTTAGATTGTCTACCTGCCTTTCTTTT 298

5m.2 TCACAGTTCACACACAAATTGTTCATTTGAGGTGTTAGATTGTCTACCTGCCTTTCTTTT 298

5m.9 TCACAGTTCGCACACAAATTGTTCATTTGAGGTGTTAGATTGTCTACCTGCCTGTCTTTT 463

WT TCACAGTTCACACACAAATTGTTCATTTGAGGTGTTAGATTGTCTACCTGCCTTTCTTTT 463

5m.10 TCACAGTTCACACACAAATTGTTCATTTGAGGTGTCAGATTGTCTACCTGCCTTTCTTTT 463

********* * *********************** **************** *****

5m.13 GCTAACAGCATACTTCTAGCAAATCTCAGCAGCAGCAATTTGTTTTGCAGGGTGTGCCGG 540

5m.19 GCTAGCCGCATACATCTAGCAAATCTTAGCCGCAGCAATTTGTTTTGCAGGGTGTGCCGG 522

5m.18b ACTAGCCGCATACATCTAGCAAATCTTAGCCGCAGCAATTTGTTTTGCAGGGTGTGCCGG 358

5m.17 GCTAGCCGCATACATCTAGCAAATCTTAGCCGCAGCAATTTGTTTTGCAGGGTGTGCCGG 358

5m.1 GCTAGCCGCATACATCTAGCAAATCTTAGCCGCAGCAATTTGTTTTGCAGGGTGTGCCGG 358

5m.16 GCTAGCCGCATACATCTAGCAAATCTTAGCCGCAGCAATTTGTTTTGCAGGGTGTGCCGG 358

5m.3 GCTAGCCGCATACATCTAGCAAATCTTAGCCGCAGCAATTTGTTTTGCAGGGTGTGCCGG 358

5m.18a GCTAGCCGCATACATCTAGCAAATCTTAGCCGCAGCAATTTGTTTTGCAGGGTGTGCCGG 358

5m.2 GCTAGCCGCATACATCTAGCAAATCTTAGCCGCAGCAATTTGTTTTGCAGGGTGTGCCGG 358

5m.9 GCTAGCCGCATACATCTAGCAAATCTTAGCCGCAGCAATTTGTTTTGCAGGGTGTGCCGG 523

WT GCTAACAGCATACTTCTAGCAAATCTCAGCAGCAGCAATTTGTTTTGCAGGGTGTGCCGG 523

5m.10 GCTAACAGCATACTTCTAGCAAATCTCAGCAGCAGCAATTTGTTTTGCAGGGTGTGCCGG 523

*** * ****** ************ *** *****************************

5m.13 ATAGGGTGACAGAT 554

5m.19 ATAGGGTGACAGAT 536

5m.18b ATAGGGTGACAGAT 372

5m.17 ATAGGGTGACAGAT 372

5m.1 ATAGGGTGACAGAT 372

5m.16 ATAGGGTGACAGAT 372

5m.3 ATAGGGTGACAGAT 372

5m.18a ATAGGGTGACAGAT 372

5m.2 ATAGGGTGACAGAT 372

5m.9 ATAGGGTGACAGAT 537

WT ATAGGGTGACAGAT 537

5m.10 ATAGGGTGACAGAT 537

**************

**Fig. S3** Multiple sequence alignment using Clustal Omega of *PDS* fragments cloned from the chimeric mutant plant PDS.5m. WT, DNA sequence of the D1 clone isolated from non-transformed ‘Désirée’. The 20-bp region corresponding to the sgRNA is in red and blue colours. The *Mly*I recognition site is in blue. Differences compared to WT are highlighted in green.

**PDS.3w WHITE plant**

PDS3.16 TTTCCCCGAAGCTTTACCCGCTCCTTTAAATGGTGAGCACATCATAAATAATCATGTTGT 60

PDS3.7 TTTCCCCGAAGCTTTACCCGCTCCTTTAAATGGTGAGCACATCATAAATAATCATGTTGT 60

WT TTTCCCCGAAGCTTTACCCGCTCCTTTAAATGGTGAGCACATCATAAATAATCATGTTGT 60

PDS3.2 TTTCCCCGAAGCTTTACCCGCTCCTTTAAATGGTGAGCACATCATAAATAATCATGTTGT 60

PDS3.15 TTTCCCCGAAGCTTTACCCGCTCCTTTAAATGGTGAGCACATCATAAATAATCATGTTGT 59

************************************************************

PDS3.16 CAAACTTCCCTAAAAGTTATTAAATTGATTATTCAATTACACCTATGGGACTTTACTAAC 120

PDS3.7 CAAACTTCCCTAAAAGTTATTAAATTGATTATTCAGTTACACCTATGGGACTTTACTAAC 120

WT CAAACTTCCCTAAAAGTTATTAAATTGATTATTCAATTACACCTATGGGACTTTACTAAC 120

PDS3.2 CAAACTTCCCTAAAAGTTATTAAATTGATTATTCAATTACACCTATGGGACTTTACTAAC 120

PDS3.15 CAAACTTCCCTAAAAGTTATTAAATTGATTATTCAATTACACCTATGGGACTTTACTAAC 119

*********************************** ************************

PDS3.16 CTTAAAAGAGCAGTAAGGTTCATTACTTCTTCATCGGACCTTTTGTGTGCAGCTAAAGTG 180

PDS3.7 CTTAAAAGAGCAGTAAGGTTCATTACTTTTTCATCGGACCTTTTGTGTGCAGCTAAAGTG 180

WT CTTAAAAGAGCAGTAAGGTTCATTACTTCTTCATCGGACCTTTTGTGTGCAGCTAAAGTG 180

PDS3.2 CTTAAAAGAGCAGTAAGGTTCATTACTTCTTCATCGGACCTTTTGTGTGCAGCTAAAGTG 180

PDS3.15 CTTAAAAGAGCAGTAAGGTTCATTACTTCTTCATCGGACCTTTTGTGTGCAGCTAAAGTG 179

**************************** *******************************

PDS3.16 TTAACTGCTTCGATATAATTTGCAGGTTACTTTTTAAGCATAAACCTGGCTGTATTGTTC 240

PDS3.7 TTAACTGCTTTGATATAATTTGCAGGAGTTTTGGCCATCCTAAAGAACAATGAAATGCTT 240

WT TTAACTGCTTTGATATAATTTGCAGGAGTTTTGGCCATCCTAAAGAACAATGAAATGCTT 240

PDS3.2 TTAACTGCTTTGATATAATTTGCAGGTTACTTTTTAAGCATAAACCTGGCTGTATTG--- 237

PDS3.15 TTAACTGCTTTGATATAATTTGCAGGTTACTTTTTAAGCATAAACCTGGCTGTATTG--- 236

********** *************** ** * * **** ** * **

PDS3.16 ACAGTTCACACACAAATTGTTC-------------------------------------- 292

PDS3.7 ACATGGCCAGAGAAAGTCAAATTTGCAATTGGACTCTTGCCAGCAATGCTTGGAGGGCAA 300

WT ACATGGCCAGAGAAAGTCAAATTTGCAATTGGACTCTTGCCAGCAATGCTTGGAGGGCAA 300

PDS3.2 ------------------------------------------------------------ 237

PDS3.15 ------------------------------------------------------------ 236

PDS3.16 ------------------------------------------------------------ 322

PDS3.7 TCTTATGTTGAAGCTCAAGACGGGATAAGTGTTAAGGACTGGATGAGAAAGCAAGTGCGT 360

WT TCTTATGTTGGGGCTCAAGATGGGATAAGTGTCAAGGACTGGATGAGAAAGCAAGTGCGT 360

PDS3.2 ------------------------------------------------------------ 237

PDS3.15 ------------------------------------------------------------ 236

PDS3.16 ------------------------------------------------GTTCACACACAA 322

PDS3.7 AATTAGTTATGTTACTTTTTAAGCATAAACCTGGCTGTATAGTTCACAGTTCACACAC-- 418

WT AAACAGTTATGTTACTTTTTAAGCATAAACCTGGCTGTATAGTTCACAGTTCACACACAA 420

PDS3.2 ------------------------------------------TTCACAGTTCACACACAA 255

PDS3.15 ------------------------------------------TTCACAGTTCACACACAA 254

PDS3.16 -------ATTTGAGGTGTTAGATTGTCTACCTGCCTTTCTTTTGCTAGCAGCATACATCT 322

PDS3.7 ATTGTTCATTTGAGGTGCTAGATTGTCTACCTGCCTTTCTTTTGCTAGCAGCATACATCT 478

WT ATTGTTCATTTGAGGTGTTAGATTGTCTACCTGCCTTTCTTTTGCTAACAGCATACTTCT 480

PDS3.2 ATTGTTCATTTGAGGTGTTAGATTGTCTACCTGCCTTTCTTTTGCTAGCAGCATACATCT 315

PDS3.15 ATTGTTCATTTGAGGTGTTAGATTGTCTACCTGCCTTTCTTTTGCTAGCCGCATACATCT 314

*******************

PDS3.16 AGCAAATCTTAGCCGCAGCAATTTGTTTTGCAGGGTGTGCCGGATAGGGTGACAGAT 373

PDS3.7 AGCAAATCTTAGCCGCAGCAATTTGTTTTGCAGGGTGTGCCGGATAGGGTGACAGAT 535

WT AGCAAATCTCAGCAGCAGCAATTTGTTTTGCAGGGTGTGCCGGATAGGGTGACAGAT 537

PDS3.2 AGCAAATCTTAGCCGCAGCAATTTGTTTTGCAGGGTGTGCCGGATAGGGTGACAGAT 372

PDS3.15 AGCAAATCTTAGCCGCAGCAATTTGTTTTGCAGGGTGTGCCGGATAGGGTGACAGAT 371

*********************************************************

**Fig. S4** Multiple sequence alignment using Clustal Omega of *PDS* fragments cloned from the albino mutant plant PDS.3w. WT, DNA sequence of the D1 clone isolated from non-transformed ‘Désirée’. The 20-bp region corresponding to the sgRNA is in red and blue colours. The *Mly*I recognition site is in blue. Differences compared to WT are highlighted in green.

**PDS.11w WHITE plant**

WT TTTCCCCGAAGCTTTACCCGCTCCTTTAAATGGTGAGCACATCATAAATAATCATGTTGT 60

11.1 TTTCCCCGAAGCTTTACCCGCTCCTTTAAATGGTGAGCACATCATAAATAATCATGTTGT 60

11.2 TTTCCCCGAAGCTTTACCCGCTCCTTTAAATGGTGAGCACATCATAAATAATCATGTTGT 60

************************************************************

WT CAAACTTCCCTAAAAGTTATTAAATTGATTATTCAATTACACCTATGGGACTTTACTAAC 120

11.1 CAAACTTCCCTAAAAGTTATTAAATTGATTATTCAATTACACCTATGGGACTTTACTAAC 120

11.2 CAAACTTCCCTAAAAGTTATTAAATTGATTATTCAATTACACCTATGGGACTTTACTAAC 120

************************************************************

WT CTTAAAAGAGCAGTAAGGTTCATTACTTCTTCATCGGACCTTTTGTGTGCAGCTAAAGTG 180

11.1 CTTAAAAGAGCAGTAAGGTTCATTACTTCTTCATCGGACCTTTTGTGTGCAGCTAAAGTG 180

11.2 CTTAAAAGAGCAGTAAGGTTCATTACTTCTTCATCGGACCTTTTGTGTGCAGCTAAAGTG 180

************************************************************

WT TTAACTGCTTTGATATAATTTGCAGGAGTTTTGGCCATCCTAAAGAACAATGAAATGCTT 240

11.1 TTAACTGCTTTGATATAATTTGCAGG---------------------------------- 240

11.2 TTAACTGCTTTGATATAATTTGCAGG---------------------------------- 240

**************************

WT ACATGGCCAGAGAAAGTCAAATTTGCAATTGGACTCTTGCCAGCAATGCTTGGAGGGCAA 300

11.1 ------------------------------------------------------------ 242

11.2 ------------------------------------------------------------ 242

WT TCTTATGTTGGGGCTCAAGATGGGATAAGTGTCAAGGACTGGATGAGAAAGCAAGTGCGT 360

11.1 ------------------------------------------------------------ 242

11.2 ------------------------------------------------------------ 242

WT AAACAGTTATGTTACTTTTTAAGCATAAACCTGGCTGTATAGTTCACAGTTCACACACAA 420

11.1 -----------TTACTTTTTAAGCATAAACCTGGCTGTATTGTTCACAGTTCACACACAA 255

11.2 -----------TTACTTTTTAAGCATAAACCTGGCTGTATTGTTCACAGTTCACACACAA 255

*************************************************

WT ATTGTTCATTTGAGGTGTTAGATTGTCTACCTGCCTTTCTTTTGCTAACAGCATACTTCT 480

11.1 ATTGTTCATTTGAGGTGTTAGATTGTCTACCTGCCTTTCTTTTGCTAGCCGCATACATCT 315

11.2 ATTGTTCATTTGAGGTGTTAGATTGTCTACCTGCCTTTCTTTTGCTAGCCGCATACATCT 315

*********************************************** * ****** ***

WT AGCAAATCTCAGCAGCAGCAATTTGTTTTGCAGGGTGTGCCGGATAGGGTGACAGAT 537

11.1 AGCAAATCTTAGCCGCAGCAATTTGTTTTGCAGGGTGGGCCGGATAGGGTGACAGAT 372

11.2 AGCAAATCTTAGCCGCAGCAATTTGTTTTGCAGGGTGTGCCGGATAGGGTGACAGAT 372

********* *** *********************** *******************

**Fig. S5** Multiple sequence alignment using Clustal Omega of *PDS* fragments cloned from the albino mutant plant PDS.11w. WT, DNA sequence of the D1 clone isolated from non-transformed ‘Désirée’. The 20-bp region corresponding to the sgRNA is in red and blue colours. The *Mly*I recognition site is in blue. Differences compared to WT are highlighted in green.

**PDS.28g GREEN plant**

WT TTTCCCCGAAGCTTTACCCGCTCCTTTAAATGGTGAGCACATCATAAATAATCATGTTGT 60

28.2 TTTCCCCGAAGCTTTACCCGCTCCTTTAAATGGTGAGCACATCATAAATAATCATGTTGT 60

28.4 TTTCCCCGAAGCTTTACCCGCTCCTTTAAATGGTGAGCACACCATAAATAATCATGTTGT 60

28.1 TTTCCCCGAAGCTTTACCCGCTCCTTTAAATGGTGAGCACATCATAAATAATCATGTTGT 60

28.5 TTTCCCCGAAGCTTTACCCGCTCCTTTAAATGGTGAGCACATCATAAATAATCATGTTGT 60

28.6 TTTCCCCGAAGCTTTACCCGCTCCTTTAAATGGTGAGCACATCATAAATAATCATGTTGT 60

28.3 TTTCCCCGAAGCTTTACCCGCTCCTTTAAATGGTGAGCACATCATAAATAATCATGTTGT 60

***************************************** ******************

WT CAAACTTCCCTAAAAGTTATTAAATTGATTATTCAATTACACCTATGGGACTTTACTAAC 120

28.2 CAAACTTCCCTAAAAGTTATTAAATTGATTATTCAATTACACCTATGGGACTTTACTAAC 120

28.4 CAAACTTCCCTAAAAGTTATTAAATCGATTATTCAATTACACCTATGGGACTTTACTAAC 120

28.1 CAAACTTCCCTAAAAGTTATTAAATTGATTATTCAATTACACCTATGGGACTTTACTAAC 120

28.5 CAAACTTCCCTAAAAGTTATTAAATTGATTATTCAATTACACCTATGGGACTTTACTAAC 120

28.6 CAAACTTCCCTAAAAGTTATTAAATTGATTATTCAATTACACCTATGGGACTTTACTAAC 120

28.3 CAAACTTCCCTAAAAGTTATTAAATTGATTATTCAATTACACCTATGGGACTTTACTAAC 120

************************* **********************************

WT CTTAAAAGAGCAGTAAGGTTCATTACTTCTTCATCGGACCTTTTGTGTGCAGCTAAAGTG 180

28.2 CTTAAAAGAGCAGTAAGGTTCATTACTTCTTCATCGGACCTTTTGTGTGCAGCTAAAGTG 180

28.4 CTTAAAAGAGCAGTAAGGTTCATTACTTCTTCATCGGACCTTTTGTGTGCAGCTAAAGTG 180

28.1 CTTAAAAGAGCAGTAAGGTTCATTACTTCTTCATCGGACCTTTTGTGTGCAGCTAAAGTG 180

28.5 CTTAAAAGAGCAGTAAGGTTCATTACTTCTTCATCGGACCTTTTGTGTGCAGCTAAAGTG 180

28.6 CTTAAAAGAGCAGTAAGGTTCATTACTTCTTCATCGGACCTTTTGTGTGCAGCTAAAGTG 180

28.3 CTTAAAAGAGCAGTAAGGTTCATTACTTCTTCATCGGACCTTTTGTGTGCAGCTAAAGTG 180

************************************************************

WT TTAACTGCTTTGATATAATTTGCAGGAGTTTTGGCCATCCTAAAGAACAATGAAATGCTT 240

28.2 TTAACTGCTTTGATATAATTTGCAGGAGTTTTGGCCATCCTAAAGAACAATGAAATGCTT 240

28.4 TTAACTGCTTTGATATAATTTGCAGGAGTTTTGGCCATCCTAAAGAACAATGAAATGCTT 240

28.1 TTAACTGCTTTGATATAATTTGCAGGAGTTTTGGCCATCCTAAAGAACAATGAAATGCTT 240

28.5 TTAACTGCTTTGATATAATTTGCAGGAGTTTTGGCCATCCTAAAGAACAATGAAATGCTT 240

28.6 TTAACTGCTTTGATATAATTTGCAGGAGTTTTGGCCATCCTAAAGAACAATGAAATGCTT 240

28.3 TTAACTGCTTTGATATAATTTGCAGGAGTTTTGGCCATCCTAAAGAACAATGAAATGCTT 240

************************************************************

WT ACATGGCCAGAGAAAGTCAAATTTGCAATTGGACTCTTGCCAGCAATGCTTGGAGGGCAA 300

28.2 ACATGGCCAGAGAAAGTCAAATTTGCAATTGGACTCTTGCCAGCAATGCTTGGAGGGCAA 300

28.4 ACATGGCCAGAGAAAGTCAAATTTGCAATA------------------------------ 270

28.1 ACATGGCCAGAGAAAGTCAAATTTGCAATA------------------------------ 270

28.5 ACATGGCCAGAGAAAGTCAAATTTGCAATA------------------------------ 270

28.6 ACATGGCCAGAGAAAGTCAAATTTGCAATA------------------------------ 270

28.3 ACATGGCCAGAGAAAGTCAAATTTGCAATA------------------------------ 270

*****************************

WT TCTTATGTTGGGGCTCAAGATGGGATAAGTGTCAAGGACTGGATGAGAAAGCAAGTGCGT 360

28.2 TCTTATGTTGAAGCTCAAGACGGGATAAGTGTCAAGGACTGGATGAGAAAGCAAGTGCGT 360

28.4 -----TGTTGAAGCTCAAGACGGGATAAGTGTTAAGGACTGGATGAGAAAGCAAGTGCGT 325

28.1 -----TGTTGAAGCTCAAGACGGGATAAGTGTTAAGGACTGGATGAGAAAGCAAGTGCGT 325

28.5 -----TGTTGAAGCTCAAGACGGGATAAGTGTTAAGGACTGGATGAGAAAGCAAGTGCGT 325

28.6 -----TGTTGAAGCTCAAGACGGGATAAGTGTTAAGGACTGGATGAGAAAGCAAGTGCGT 325

28.3 -----TGTTGAAGCTCAAGACGGGATAAGTGTTAAGGACTGGATGAGAAAGCAAGTGCGT 325

***** ******** *********** ***************************

WT AAACAGTTATGTTACTTTTTAAGCATAAACCTGGCTGTATAGTTCACAGTTCACACACAA 420

28.2 AAACAGTTATGTTACTTTTTAAGCATAAACCTGGCTGTATTGTTCACAGTTCACACACAA 420

28.4 AATTAGTTATGTTACTTTTTAAGCATAAACCTGGCTGTATTGTTCACAGTTCACACACAA 385

28.1 AATTAGTTATGTTACTTTTTAAGCATAAACCTGGCTGTATTGTTCACAGTTCACACACAA 385

28.5 AATTAGTTATGTTACTTTTTAAGCATAAACCTGGCTGTATTGTTCACAGTTCACACACAA 385

28.6 AATTAGTTATGTTACTTTTTAAGCATAAACCTGGCTGTATTGTTCACAGTTCACACACAA 385

28.3 AATTAGTTATGTTACTTTTTAAGCATAAACCTGGCTGTATTGTTCACAGTTCACACACAA 385

** ************************************ *******************

WT ATTGTTCATTTGAGGTGTTAGATTGTCTACCTGCCTTTCTTTTGCTAACAGCATACTTCT 480

28.2 ATTGTTCATTTGAGGTGTTAGATTGTCTACCTGCCTTTCTTTTGCTAACAGCATACATCT 480

28.4 ATTGTTCATTTGAGGTGTTAGATTGTCTACCTGCCTTTCTTTTGCTAGCCGCATACATCT 445

28.1 ATTGTTCATTTGAGGTGTTAGATTGTCTACCTGCCTTTCTTTTGCTAGCCGCATACATCT 445

28.5 ATTGTTCATTTGAGGTGTTAGATTGTCTACCTGCCTTTCTTTTGCTAGCCGCATACATCT 445

28.6 ATTGTTCATTTGAGGTGTTAGATTGTCTACCTGCCTTTCTTTTGCTAGCCGCATACATCT 445

28.3 ATTGTTCATTTGAGGTGTTAGATTGTCTACCTGCCTTTCTTTTGCTAGCCGCATACATCT 445

*********************************************** * ****** ***

WT AGCAAATCTCAGCAGCAGCAATTTGTTTTGCAGGGTGTGCCGGATAGGGTGACAGAT 537

28.2 AGCAAATCTTAGCAGCAGCAATTTGTTTTGCAGGGTGTGCCGGATAGGGTGACAGAT 537

28.4 AGCAAATCTTAGCCGCAGCAATTTGTTTTGCAGGGTGTGCCGGATAGGGTGACAGAT 502

28.1 AGCAAATCTTAGCCGCAGCAATTTGTTTTGCAGGGTGTGCCGGATAGGGTGACAGAT 502

28.5 AGCAAATCTTAGCCGCAGCAATTTGTTTTGCAGGGTGTGCCGGATAGGGTGACAGAT 502

28.6 AGCAAATCTTAGCCGCAGCAATTTGTTTTGCAGGGTGTGCCGGATAGGGTGACAGAT 502

28.3 AGCAAATCTTAGCCGCAGCTATTTGTTTTGCAGGGTGTGCCGGATAGGGTGACAGAT 502

********* *** ***** *************************************

**Fig. S6** Multiple sequence alignment using Clustal Omega of *PDS* fragments cloned from the green mutant plant PDS.28g. WT, DNA sequence of the D1 clone isolated from non-transformed ‘Désirée’. The 20-bp region corresponding to the sgRNA is in red and blue colours. The *Mly*I recognition site is in blue. Differences compared to WT are highlighted in green.

**PDS.34g GREEN plant**

34.1 TTTCCCCGAAGCTTTACCCGCTCCTTTAAATGGTGAGCACATCATAAATAATCATGTTGT 60

34.2 TTTCCCCGAAGCTTTACCCGCTCCTTTAAATGGTGAGCACATCATAAATAATCATGTTGT 60

34.5 TTTCCCCGAAGCTTTACCCGCTCCTTTAAATGGTGAGCACATCATAAATAATCATGTTGT 60

34.6 TTTCCCCGAAGCTTTACCCGCTCCTTTAAATGGTGAGCACATCATAAATAATCATGTTGT 60

WT TTTCCCCGAAGCTTTACCCGCTCCTTTAAATGGTGAGCACATCATAAATAATCATGTTGT 60

34.3 TTTCCCCGAAGCTTTACCCGCTCCTTTAAATGGTGAGCACATCATAAATAATCATGTTGT 60

************************************************************

34.1 CAAACCTCCCTAAAAGTTATTAAATTGATTATTCAATTACACCTATGGGACTTTACTAAC 120

34.2 CAAACCTCCCTAAAAGTTATTAAATTGATTATTCAATTACACCTATGGGACTTTACTAAC 120

34.5 CAAACCTCCCTAAAAGTTATTAAATTGATTATTCAATTACACCTATGGGACTTTACTAAC 120

34.6 CAAACTTCCCTAAAAGTTATTAAATTGATTATTCAATTACACCTATGGGACTTTACTAAC 120

WT CAAACTTCCCTAAAAGTTATTAAATTGATTATTCAATTACACCTATGGGACTTTACTAAC 120

34.3 CAAACTTCCCTAAAAGTTATTAAATTGATTATTCAATTACACCTATGGGACTTTACTAAC 120

***** ******************************************************

34.1 CTTAAAAGAGCAGTAAGGTTCATTACTTCTTCATCGGACCTTTTGTGTGCAGCTAAAGTG 180

34.2 CTTAAAAGAGCAGTAAGGTTCATTACTTCTTCATCGGACCTTTTGTGTGCAGCTAAAGTG 180

34.5 CTTAAAAGAGCAGTAAGGTTCATTACTTCTTCATCGGACCTTTTGTGTGCAGCTAAAGTG 180

34.6 CTTAAAAGAGCAGCAAGGTTCATTACTTCTTCATCGGACCTTTTGTGTGCAGCTAAAGTG 180

WT CTTAAAAGAGCAGTAAGGTTCATTACTTCTTCATCGGACCTTTTGTGTGCAGCTAAAGTG 180

34.3 CTTAAAAGAGCAGTAAGGTTCATTACTTTTTCATCGGACCTTTTGTGTGCAGCTAAAGTG 180

************* ************** *******************************

34.1 TTAACTGCTTTGATATAATTTGCAGGTTACTTTTTAAGCATAAACCTGGCTGTATTGTT- 239

34.2 TTAACTGCTTTGATATAATTTGCAGGTTACTTTTTAAGCATAAACCTGGCTGTATTGTT- 239

34.5 TTAACTGCTTTGATATAATTTGCAGGTTACTTTTTAAGCATAAACCTGGCTGTATTGTT- 239

34.6 TTAACTGCTTTGATATAATTTGCAGGTTACTTTTTAAGCATAAACCTGGCTGTATTG**A**T- 239

WT TTAACTGCTTTGATATAATTTGCAGGAGTTTTGGCCATCCTAAAGAACAATGAAATGCTT 240

34.3 TTAACTGCTTTGATATAATTTGCAGGAGTTTTGGCCATCCTAAAGAACAATGAAATGCTT 240

************************** ** * * **** ** * ** *

34.1 ------------------------------------------------------------ 239

34.2 ------------------------------------------------------------ 239

34.5 ------------------------------------------------------------ 239

34.6 ------------------------------------------------------------ 239

WT ACATGGCCAGAGAAAGTCAAATTTGCAATTGGACTCTTGCCAGCAATGCTTGGAGGGCAA 300

34.3 ACATGGCCAGAGAAAGTCAAATTTGCAATTGGACTCTTGCCAGCAATGCTTGGAGGGCAA 300

34.1 ------------------------------------------------------------ 239

34.2 ------------------------------------------------------------ 239

34.5 ------------------------------------------------------------ 239

34.6 ------------------------------------------------------------ 239

WT TCTTATGTTGGGGCTCAAGATGGGATAAGTGTCAAGGACTGGATGAGAAAGCAAGTGCGT 360

34.3 TCTTATGTTGAAGCTCAAGACGGGATAAGTGTTAAGGACTGGATGAGAAAGCAAGTGCGT 360

34.1 --------------------------------------------CACAGTTCACACACAA 255

34.2 --------------------------------------------CACAGTTCACACACAA 255

34.5 --------------------------------------------CACAGTTCACACACAA 255

34.6 --------------------------------------------CACAGTTCACACACAA 255

WT AAACAGTTATGTTACTTTTTAAGCATAAACCTGGCTGTATAGTTCACAGTTCACACACAA 420

34.3 AATTAGTTATGTTACTTTTTAAGCATAAACCTGGCTGTATAGTTCACAGTTCACAC--AC 418

************ *

34.1 ATTGTTCATTTGAGGTGTTAGATTGTCTACCTGCCTTTCTTTTGCTAGCCGCATACATCT 315

34.2 ATTGTTCATTTGAGGTGTTAGATTGTCTACCTGCCTTTCTTTTGCTAGCCGCATACATCT 315

34.5 ATTGTTCATTTGAGGTGTTAGATTGTCTACCTGCCTTTCTTTTGCTAGCCGCATACATCT 315

34.6 ATTGTTCATTTGAGGTGTTAGATTGTCTACCTGCCTTTCTTTTGCTAGCCGCATACATCT 315

WT ATTGTTCATTTGAGGTGTTAGATTGTCTACCTGCCTTTCTTTTGCTAACAGCATACTTCT 480

34.3 ATTGTTCATTTGAGGTGTTAGATTGTCTACCTGCCTTTCTTTTGCTAGCAGCATACATCT 478

*********************************************** * ****** ***

34.1 AGCAAATCTTAGCCGCAGCAATTTGTTTTGCAGGGTGTGCCGGATAGGGTGACAGAT 372

34.2 AGCAAATCTTAGCCGCAGCAATTTGTTTTGCAGGGTGTGCCGGATAGGGTGACAGAT 372

34.5 AGCAAATCTTAGCCGCAGCAATTTGTTTTGCAGGGTGTGCCGGATAGGGTGACAGAT 372

34.6 AGCAAATCTTAGCCGCAGCAATTTGTTTTGCAGGGTGTGCCGGATAGGGTGACAGAT 372

WT AGCAAATCTCAGCAGCAGCAATTTGTTTTGCAGGGTGTGCCGGATAGGGTGACAGAT 537

34.3 AGCAAATCTTAGCCGCAGCAATTTGTTTTGCAGGGTGTGCCGGATAGGGTGACAGAT 535

********* *** *******************************************

**Fig. S7** Multiple sequence alignment using Clustal Omega of *PDS* fragments cloned from the green mutant plant PDS.34g. WT, DNA sequence of the D1 clone isolated from non-transformed ‘Désirée’. The 20-bp region corresponding to the sgRNA is in red and blue colours. The *Mly*I recognition site is in blue. Differences compared to WT are highlighted in green.

**PDS.73g GREEN plant**

73.4 -TTTCCCCGAAGCTTTACCCGCTCCTTTAAATGGTGAGCACATCATGAATAAATTTAGCC 59

WT -TTTCCCCGAAGCTTTACCCGCTCCTTTAAATGGTGAGCACATCATAAATAAT------- 52

73.8 -TTTCCCCGAAGCTTTACCCGCTCCTTTAAATGGTGAGCACATCATAAATAAT------- 52

73.9 TTTTCCCCGAAGCTTTACCCGCTCCTTTAAATGGTGAGCACATCATAAATAAT------- 53

73.11 -TTTCCCCGAAGCTTTACCCGCTCCTTTAAATGGTGAGCACATCATAAATAAT------- 52

73.2 TTTTCCCCGAAGCTTTACCCGCTCCTTTAAATGGTGAGCACATCATAAATAAT------- 53

73.12 -TTTCCCCGAAGCTTTACCCGCTCCTTTAAATGGTGAGCACATCATAAATAAT------- 52

********************************************* *****

73.4 CTCTGTAATCCTGTTGTCAAACTTCCCTAATAAGTTATTAAATTGATTATTCAATTACAC 119

WT ---------CATGTTGTCAAACTTCCCTAAAA-GTTATTAAATTGATTATTCAATTACAC 102

73.8 ---------CATGTTGTCAAACTTCCCTAAAA-GTTATTAAATTGATTATTCAATTACAC 102

73.9 ---------CATGTTGTCAAACTTCCCTAAAA-GTTATTAAATTGATTATTCAATTACAC 103

73.11 ---------CATGTTGTCAAACTTCCCTAAAA-GTTATTAAATTGATTATTCAATTACAC 102

73.2 ---------CATGTTGTCAAACTTCCCTAAAA-GTTATTAAATTGATTATTCAATTACAC 103

73.12 ---------CATGTTGTCAAACTTCCCTAAAA-GTTATTAAATTGATTATTCAATTACAC 102

* ******************* * ***************************

73.4 CTTTGGGACTTTACTAACCTTAAAAGAGCATTAAGGTTCATTACTTCTTCATCGGACCTT 179

WT CTATGGGACTTTACTAACCTTAAAAGAGCAGTAAGGTTCATTACTTCTTCATCGGACCTT 162

73.8 CTATGGGACTTTACTAACCTTAAAAGAGCAGTAAGGTTCATTACTTCTTCATCGGACCTT 162

73.9 CTATGGGACTTTACTAACCTTAAAAGAGCAGTAAGGTTCATTACTTCTTCATCGGACCTT 163

73.11 CTATGGGACTTTACTAACCTTAAAAGAGCAGTAAGGTTCATTACTTCTTCATCGGACCTT 162

73.2 CTATGGGACTTTACTAACCTTAAAAGAGCAGTAAGGTTCATTACTTTTTCATCGGACCTT 163

73.12 CTATGGGACTTTACTAACCTTAAAAGAGCAGTAAGGTTCATTACTTTTTCATCGGACCTT 162

** *************************** *************** *************

73.4 TTGTGTGCAGCTAAAGTGTTAACTGCTTCGATATAATTTGCAGGAGTTTTGGCCATCCTA 239

WT TTGTGTGCAGCTAAAGTGTTAACTGCTTTGATATAATTTGCAGGAGTTTTGGCCATCCTA 222

73.8 TTGTGTGCAGCTAAAGTGTTAACTGCTTTGATATAATTTGCAGGAGTTTTGGCCATCCTA 222

73.9 TTGTGTGCAGCTAAAGTGTTAACTGCTTTGATATAATTTGCAGGAGTTTTGGCCATCCTA 223

73.11 TTGTGTGCAGCTAAAGTGTTAACTGCTTTGATATAATTTGCAGGAGTTTTGGCCATCCTA 222

73.2 TTGTGTGCAGCTAAAGTGTTAACTGCTTTGATATAATTTGCAGGAGTTTTGGCCATCCTA 223

73.12 TTGTGTGCAGCTAAAGTGTTAACTGCTTTGATATAATTTGCAGGAGTTTTGGCCATCCTA 222

**************************** *******************************

73.4 AAGAACAATGAAATGCTTACATGGCCAGAGAAAGTCAAATTTGCAATTG-ACTCTTGCCA 299

WT AAGAACAATGAAATGCTTACATGGCCAGAGAAAGTCAAATTTGCAATTGGACTCTTGCCA 282

73.8 AAGAACAATGAAATGCTTACATGGCCAGAGAAAGTCAAATTTGCAATTGGACTCTTGCCA 282

73.9 AAGAACAATGAAATGCTTACATGGCCAGAGAAAGTCAAATTTGCAATTGGACTCTTGCCA 283

73.11 AAGAACAATGAAATGCTTACATGGCCAGAGAAAGTCAAATTTGCAATTGGACTCTTGCCA 282

73.2 AAGAACAATGAAATGCTTACATGGCCAGAGAAAGTCAAATTTGCAATTGGACTCTTGCCA 283

73.12 AAGAACAATGAAATGCTTACATGGCCAGAGAAAGTCAAATTTGCAATTGGACTCTTGCCA 282

************************************************************

73.4 GCAATGCTTGGAGGGCAATCTTATGTTGAGGCTCAAGATGGGATAAGTGTCAAGGACTGG 359

WT GCAATGCTTGGAGGGCAATCTTATGTTGGGGCTCAAGATGGGATAAGTGTCAAGGACTGG 342

73.8 GCAATGCTTGGAGGGCAATCTTATGTTGAAGCTCAAGACAGGATAAGTGTTAAGGACTGG 342

73.9 GCAATGCTTGGAGGGCAATCTTATGTTGAAGCTCAAGACGGGATAAGTGTTAAGGACTGG 343

73.11 GCAATGCTTGGAGGGCAATCTTATGTTGAAGCTCAAGACGGGATAAGTGTTAAGGACTGG 342

73.2 GCAATGCTTGGAGGGCAATCTTATGTTGAAGCTCAAGACGGGATAAGTGTTAAGGACTGG 343

73.12 GCAATGCTTGGAGGGCAATCTTATGTTGAAGCTCAAGACGGGATAAGTGTTAAGGACTGG 342

**************************** ******** ********** *********

73.4 ATGAGAAAGCAAGTGCGTAATCAGTTATGTTACTTTTTAAGCATAAACCTGGCTGTATAG 419

WT ATGAGAAAGCAAGTGCGTAAACAGTTATGTTACTTTTTAAGCATAAACCTGGCTGTATAG 402

73.8 ATGAGAAAGCAAGTGCGTAATTAGTTATGTTACTTTTTAAGCATAAACCTGGCTGTATTG 402

73.9 ATGAGAAAGCAAGTGCGTAATTAGTTATGTTACTTTTTAAGCATAAACCTGGCTGTATTG 403

73.11 ATGAGAAAGCAAGTGCGTAATTAGTTATGTTACTTTTTAAGCATAAACCTGGCTGTATTG 402

73.2 ATGAGAAAGCAAGTGCGTAATTAGTTATGTCACTTTTTAAGCATAAACCTGGCTGTATAG 403

73.12 ATGAGAAAGCAAGTGCGTAATTAGTTATGTTACTTTTTAAGCATAAACCTGGCTGTATAG 402

******************** ******** *************************** *

73.4 TTCACAGTTCACACACAAATTGTTCATTTGAGGTGTTAGATTGTCTACCTGCCTTTCTTT 479

WT TTCACAGTTCACACACAAATTGTTCATTTGAGGTGTTAGATTGTCTACCTGCCTTTCTTT 462

73.8 TTCACAGTTCACACACAAATTGTTCATTTGAGGTGTTAGATTGTCTACCTGCCTTTCTTT 462

73.9 TTCACAGTTCACACACAAATTGTTCATTTGAGGTGTTAGATTGTCTACCTGCCTTTCTTT 463

73.11 TTCACAGTTCACACACAAATTGTTCATTTGAGGTGTTAGATTGTCTACCTGCCTTTCTTT 462

73.2 TTCACAGTTCACAC--ACATTGTTCATTTGAGGTGTTAGATTGCCTACCTGCCTTTCTTT 461

73.12 TTCACAGTTCACAC--ACATTGTTCATTTGAGGTGTTAGATTGTCTACCTGCCTTTCTTT 460

************** * ************************* ****************

73.4 TGCTAACAGCATACTTCTAGCAAATCTCAGCAGCAGCAATTTGTTTTGCAGGGTGTGCCG 539

WT TGCTAACAGCATACTTCTAGCAAATCTCAGCAGCAGCAATTTGTTTTGCAGGGTGTGCCG 522

73.8 TGCTAGCCGCATACATCTAGCAAATCTTAGCCGCAGCAATTTGTTTTGCAGGGTGTGCCG 522

73.9 TGCTAGCCGCATACATCTAGCAAATCTTAGCCGCAGCAATTTGTTTTGCAGGGTGTGCCG 523

73.11 TGCTAGCCGCATACATCTAGCAAATCTTAGCCGCAGCAATTTGTTTTGCAGGGTGTGCCG 522

73.2 TGCTAGCAGCATACATCTAGCAAATCTTAGCCGCAGCAATTTGTTTTGCAGGGTGTGCCG 521

73.12 TGCTAGCAGCATACATCTAGCAAATCTTAGCCGCAGCAATTTGTTTTGCAGGGTGTGCCG 520

***** * ****** ************ *** ****************************

73.4 GATAGGGTGACAGAT 554

WT GATAGGGTGACAGAT 537

73.8 GATAGGGTGACAGAT 537

73.9 GATAGGGTGACAGAT 538

73.11 GATAGGGTGACAGAT 537

73.2 GATAGGGTGACAGAT 536

73.12 GATAGGGTGACAGAT 535

***************

**Fig. S8** Multiple sequence alignment using Clustal Omega of *PDS* fragments cloned from the green mutant plant PDS.73g. WT, DNA sequence of the D1 clone isolated from non-transformed ‘Désirée’. The 20-bp region corresponding to the sgRNA is in red and blue colours. The *Mly*I recognition site is in blue. Differences compared to WT are highlighted in green.

**PDS.65g GREEN plant**

65.3 TTTCCCCGAAGCTTTACCCGCTCCTTTAAATGGTGAGCACATCATAAATAATCATGTTG 59

65.1 TTTCCCCGAAGCTTTACCCGCTCCTTTAAATGGTGAGCACATCATAAATAATCATGTTG 59

65.2 TTTCCCCGAAGCTTTACCCGCTCCTTTAAATGGTGAGCACATCATAAATAATCATGTTG 59

65.5 TTTCCCCGAAGCTTTACCCGCTCCTTTAAATGGTGAGCACATCATAAATAATCATGTTG 59

WT TTTCCCCGAAGCTTTACCCGCTCCTTTAAATGGTGAGCACATCATAAATAATCATGTTG 59

65.6 TTTCCCCGAAGCTTTACCCGCTCCTTTAAATGGTGAGCACATCATAAATAATCATGTTG 60

***********************************************************

65.3 TCAAACTTCCCTAAAAGTTATTAAATTGATTATTCAATTACACCTATGGGACTTTACTAA 119

65.1 TCAAACTTCCCTAAAAGTTATTAAATTGATTATTCAATTACACCTATGGGACTTTACTAA 119

65.2 TCAAACTTCCCTAAAAGTTATTAAATTGATTATTCAATTACACCTATGGGACTTTACTAA 119

65.5 TCAAGCTTCCCTAAAAGTTATTAAATTGATTATTCAATTACACCTATGGGACTTTACTAA 119

WT TCAAACTTCCCTAAAAGTTATTAAATTGATTATTCAATTACACCTATGGGACTTTACTAA 119

65.6 TCAAACTTCCCTAAAAGTTATTAAATTGATTATTCAATTACACCTATGGGACTTTACTAA 120

**** *******************************************************

65.3 CCTTAAAAGAGCAGTAAGGTTCATTACTTTTTCATCGGACCTTTTGTGTGCAGCTAAAGT 179

65.1 CCTTAAAAGAGCAGTAAGGTTCATTACTTTTTCATCGGACCTTTTGTGTGCAGCTAAAGT 179

65.2 CCTTAAAAGAGCAGTAAGGTTCATTACTTCTTCATCGGACCTTTTGTGTGCAGCTAAAGT 179

65.5 CCTTAAAAGAGCAGTAAGGTTCATTACTTCTTCATCGGACCTTTTGTGTGCAGCTAAAGT 179

WT CCTTAAAAGAGCAGTAAGGTTCATTACTTCTTCATCGGACCTTTTGTGTGCAGCTAAAGT 179

65.6 CCTTAAAAGAGCAGTAAGGTTCATTACTTCTTCATCGGACCTTTTGTGTGCAGCTAAAGT 180

***************************** ******************************

65.3 GTTAACTGCTTTGATATAATTTGCAGGAGTTTTGGCCATCCTAAAGAACAATGAAATGCT 239

65.1 GTTAACTGCTTTGATATAATTTGCAGGAGTTTTGGCCATCCTAAAGAACAATGAAATGCT 239

65.2 GTTAACTGCTTTGATATAATTTGCAGGAGTTTTGGCCATCCTAAAGAACAATGAAGTGCT 239

65.5 GTTAACTGCTTTGATATAATTTGCAGGAGTTTTGGCCATCCTAAAGAACAATGAAATGCT 239

WT GTTAACTGCTTTGATATAATTTGCAGGAGTTTTGGCCATCCTAAAGAACAATGAAATGCT 239

65.6 GTTAACTGCTTTGATATAATTTGCAGGAGTTTTGGCCATCCTAAAGAACAATGAAATGCT 240

******************************************************* ****

65.3 TACATGGCCAGAGAAAGTCAAATTTGCAATTGGACTCTTGCCAGCAATGCTTGGAGGGCA 299

65.1 TACATGGCCAGAGAAAGTCAAATTTGCAATTG-ACTCTTGCCAGCAATGCTTGGAGGGCA 299

65.2 TACATGGCCAGAGAAAGTCAAATTTGCAATTGGACTCTTGCCAGCAATGCTTGGAGGGCA 299

65.5 TACATGGCCAGAGAAAGTCAAATTTGCAATTGGACTCTTGCCAGCAATGCTTGGAGGGCA 299

WT TACATGGCCAGAGAAAGTCAAATTTGCAATTGGACTCTTGCCAGCAATGCTTGGAGGGCA 299

65.6 TACATGGCCAGAGAAAGCCAAATTTGCAATTGGACTCTTGCCAGCAATGCTTGGAGGGCA 300

***************** ******************************************

65.3 ATCTTATGTTGAAGCTCAAGACGGGATAAGTGTTAAGGACTGGATGAGAAAGCAAGTGCG 359

65.1 ATCTTATGTTGAAGCTCAAGACGGGATAAGTGTTAAGGACTGGATGAGAAAGCAAGTGCG 359

65.2 ATCTTATGTTGAAGCTCAAGACGGGATAAGTGTTAAGGACTGGATGAGAAAGCAAGTGCG 359

65.5 ATCTTATGTTGAAGCTCAAGACGGGATAAGTGCTAAGGACTGGATGAGAAAGCAAGTGCG 359

WT ATCTTATGTTGGGGCTCAAGATGGGATAAGTGTCAAGGACTGGATGAGAAAGCAAGTGCG 359

65.6 ATCTTATGTTGAGGCTCAAGATGGGATAAGTGTCAAGGACTGGATGAGAAAGCAAGTGCG 360

*********** ******** ********** **************************

65.3 TAATTAGTTATGTTACTTTTTAAGCATAAACCTGGCTGTATAGTTCACAGTTCACAC--A 417

65.1 TAATTAGTTATGTTACTTTTTAAGCATAAACCTGGCTGTATTGTTCACAGTTCACACACA 419

65.2 TAATTAGTTATGTTACTTTTTAAGCATAAACCTGGCTGTATTGTTCACAGTTCACACACA 419

65.5 TAATTAGTTATGTTACTTTTTAAGCATAAACCTGGCTGTATTGTTCACAGTTCACACACA 419

WT TAAACAGTTATGTTACTTTTTAAGCATAAACCTGGCTGTATAGTTCACAGTTCACACACA 419

65.6 TAATCAGTTATGTTACTTTTTAAGCATAAACCTGGCTGTATAGTTCACAGTTCACACACA 420

*** ************************************ *************** *

65.3 CATTGTTCATTTGAGGTGTTAGATTGTCTACCTGCCTTTCTTTTGCTTGCAGCATACATC 477

65.1 AATTGTTCATTTGAGGTGTTAGATTGTCTACCTGCCTTTCTTTTGCTAGCCGCATACATC 479

65.2 AATTGTTCATTTGAGGTGTTAGATTGTCTACCTGCCTTTCTTTTGCTAGCCGCATACATC 479

65.5 AATTGTTCATTTGAGGTGTTAGATTGTCTACCTGCCTTTCTTTTGCTAGCCGCATACATC 479

WT AATTGTTCATTTGAGGTGTTAGATTGTCTACCTGCCTTTCTTTTGCTAACAGCATACTTC 479

65.6 AATTGTTCATTTGAGGTGTTAGATTGTCTACCTGCCTTTCTTTTGCTAGCAGCATACTTC 480

********************************************** * ****** **

65.3 TAGCAAATCTTAGCCGCAGCAATTTGTTTTGCAGGGTGTGCCGGATAGGGTGACAGAT 535

65.1 TAGCAAATCTTAGCCGCAGCAATTTGTTTTGCAGGGTGTGCCGGATAGGGTGACAGAT 537

65.2 TAGCAAATCTTAGCCGCAGCAATTTGTTTTGCAGGGTGTGCCGGATAGGGTGACAGAT 537

65.5 TAGCAAATCTTAGCCGCAGCAATTTGTTTTGCAGGGTGTGCCGGATAGGGTGACAGAT 537

WT TAGCAAATCTCAGCAGCAGCAATTTGTTTTGCAGGGTGTGCCGGATAGGGTGACAGAT 537

65.6 TAGCAAATCTTAGCAGCAGCAATTTGTTTTGCAGGGTGTGCCGGATAGGGTGACAGAT 538

********** *** *******************************************

**Fig. S9** Multiple sequence alignment using Clustal Omega of *PDS* fragments cloned from the green mutant plant PDS.65g. WT, DNA sequence of the D1 clone isolated from non-transformed ‘Désirée’. The 20-bp region corresponding to the sgRNA is in red and blue colours. The *Mly*I recognition site is in blue. Differences compared to WT are highlighted in green.

**PDS.48g GREEN plant**

WT TTTCCCCGAAGCTTTACCCGCTCCTTTAAATGGTGAGCACATCATAAATAATCATGTTGT 60

48.3 TTTCCCCGAAGCTTTACCCGCTCCTTTAAATGGTGAGCACATCATAAATAATCATGTTGT 60

48.7 TTTCCCCGAAGCTTTACCCGCTCCTTTAAATGGTGAGCACATCATAAATAATCATGTTGT 60

48.1 TTTCCCCGAAGCTTTACCCGCTCCTTTAAATGGTGAGCACATCATAAATAATCATGTTGT 60

48.5 TTTCCCCGAAGCTTTACCCGCTCCTTTAAATGGTGAGCACATCATAAATAATCATGTTGT 60

************************************************************

WT CAAACTTCCCTAAAAGTTATTAAATTGATTATTCAATTACACCTATGGGACTTTACTAAC 120

48.3 CAAACTTCCCTAAAAGTTATTAAATTGATTATTCAATTACACCTATGGGACTTTACTAAC 120

48.7 CAAACTTCCCTAAAAGTTATTAAATTGATTATTCAATTACACCTATGGGACTTTACTAAC 120

48.1 CAAACTTCCCTAAAAGTTATTAAATTGATTATTCAATTACACCTATGGGACTTTACTAAC 120

48.5 CAAACTTCCCTAAAAGTTATTAAATTGATTATTCAATTACACCTATGGGACTTTACTAAC 120

************************************************************

WT CTTAAAAGAGCAGTAAGGTTCATTACTTCTTCATCGGACCTTTTGTGTGCAGCTAAAGTG 180

48.3 CTTAAAAGAGCAGTAAGGTTCATTACTTTTTCATCGGACCTTTTGTGTGCAGCTAAAGTG 180

48.7 CTTAAAAGAGCAGTAAGGTTCATTACTTTTTCATCGGACCTTTTGTGTGCAGCTAAAGTG 180

48.1 CTTAAAAGAGCAGTAAGGTTCATTACTTCTTCATCGGACCTTTTGTGTGCAGCTAAAGTG 180

48.5 CTTAAAAGAGCAGTAAGGTTCATTACTTCTTCATCGGACCTTTTGTGTGCAGCTAAAGTG 180

**************************** *******************************

WT TTAACTGCTTTGATATAATTTGCAGGAGTTTTGGCCATCCTAAAGAACAATGAAATGCTT 240

48.3 TTAACTGCTTTGATATAATTTGCAGGAGTTTTGGCCATCCTAAAGAACAATGAAATGCTT 240

48.7 TTAACTGCTTTGATATAATTTGCAGGAGTTTTGGCCATCCTAAAGAACAATGAAATGCTT 240

48.1 TTAACTGCTTTGATATAATTTGCAGGAGTTTTGGCCATCCTAAAGAACAATGAAATGCTT 240

48.5 TTAACTGCTTTGATATAATTTGCAGGAGTTTTGGCCATCCTAAAGAACAATGAAATGCTT 240

************************************************************

WT ACATGGCCAGAGAAAGTCAAATTTGCAATTGGACTCTTGCCAGCAATGCTTGGAGGGCAA 300

48.3 ACATGGCCAGAGAAAGTCAAATTTGCAATTGGACTCTTGCCAGCAATGCTTGGAGGGCAA 300

48.7 ACATGGCCAGAGAAAGTCAAATTTGCAATTGGACTCTTGCCAGCAATGCTTGGAGGGCAA 300

48.1 ACATGGCCAGAGAAAGTCAAATTTGCAATTGGACTCTTGCCAGCAATGCTTGGAGGGCAA 300

48.5 ACATGGCCAGAGAAAGTCAAATTTGCAAT**A--**ACTCTTGCCAGCAATGCTTGGAGGGCAA 300

************************************************************

WT TCTTATGTTGGGGCTCAAGATGGGATAAGTGTCAAGGACTGGATGAGAAAGCAAGTGCGT 360

48.3 TCTTATGTTGAAGCTCAAGACGGGATAAGTGTTAAGGACTGGATGAGAAAGCAAGTGCGT 360

48.7 TCTTATGTTGAAGCTCAAGACGGGATAAGTGTTAAGGACTGGATGAGAAAGCAAGTGCGT 360

48.1 TCTTATGTTGAAGCTCAAGACGGGATAAGTGTTAAGGACTGGATGAGAAAGCAAGTGCGT 360

48.5 TCTTATGTTGAAGCTCAAGACGGGATAAGTGTTAAGGACTGGATGAGAAAGCAAGTGCGT 360

********** ******** *********** ***************************

WT AAACAGTTATGTTACTTTTTAAGCATAAACCTGGCTGTATAGTTCACAGTTCACACACAA 420

48.3 AATTAGTTATGTTACTTTTTAAGCATAAACCTGGCTGTATAGTTCACAGTTCACAC--AC 418

48.7 AATTAGTTATGTTACTTTTTAAGCATAAACCTGGCTGTATAGTTCACAGTTCACAC--AC 418

48.1 AATTAGTTATGTTACTTTTTAAGCATAAACCTGGCTGTATTGTTCACAGTTCACACACAA 420

48.5 AATTAGTTATGTTACTTTTTAAGCATAAACCTGGCTGTATTGTTCACAGTTCACACACAA 420

** ************************************ *************** *

WT ATTGTTCATTTGAGGTGTTAGATTGTCTACCTGCCTTTCTTTTGCTAACAGCATACTTCT 480

48.3 ATTGTTCATTTGAGGTGTTAGATTGTCTACCTGCCTTTCTTTTGCTAGCAGCATACATCT 478

48.7 ATTGTTCATTTGAGGTGTTAGATTGTCTACCTGCCTTTCTTTTACTAGCAGCATACATCT 478

48.1 ATTGTTCATTTGAGGTGTTAGATTGTCTACCTGCCTTTCTTTTGCTAGCCGCATACATCT 480

48.5 ATTGTTCATTTGAGGTGTTAGATTGTGTACCTGCCTTTCTTTTGCTAGCCGCNTTCATCT 480

************************** **************** *** * ** * * ***

WT AGCAAATCTCAGCAGCAGCAATTTGTTTTGCAGGGTGTGCCGGATAGGGTGACAGAT 537

48.3 AGCAAATCTTAGCCGCAGCAATTTGTTTTGCAGGGTGTGCCGGATAGGGTGACAGAT 535

48.7 AGCAAATCTTAGCCGCAGCAATTTGTTTTGCAGGGTGTGCCGGATAGGGTGACAGAT 535

48.1 AGCAAATCTTAGCCGCAGCAATTTGTTTTGCAGGGTGTGCCGGATAGGGTGACAGAT 537

48.5 AGCAAATCTTAGCCGCAGCAATTTGTTTTGCAGGGTGTGCCGGATAGGGTGACAGAT 537

********* *** *******************************************

**Fig. S10** Multiple sequence alignment using Clustal Omega of *PDS* fragments cloned from the green mutant plant PDS.48g. WT, DNA sequence of the D1 clone isolated from non-transformed ‘Désirée’. The 20-bp region corresponding to the sgRNA is in red and blue colours. The *Mly*I recognition site is in blue. Differences compared to WT are highlighted in green.

**PDS.9m MOSAIC plant „tabby”**

WT TTTCCCCGAAGCTTTACCCGCTCCTTTAAATGGTGAGCACATCATAAATAAT-------- 52

Ci11 TTTCCCCGAAGCTTTACCCGCTCCTTTAAATGGTGAGCACATCATAAATAAT-------- 52

Ci12 TTTCCCCGAAGCTTTACCCGCTCCTTTAAATGGTGAGCACATCATAAATAAT-------- 52

Ci13 TTTCCCCGAAGCTTTACCCGCTCCTTTAAATGGTGAGCACATCATGAATAAATTTAGCCC 60

Ci14 TTTCCCCGAAGCTTTACCCGCTCCTTTAAATGGTGAGCACATCATGAATAAATTTAGCCC 60

Ci15 TTTCCCCGAAGCTTTACCCGCTCCTTTAAATGGTGAGCACATCATGAATAAATTTAGCCC 60

Ci16 TTTCCCCGAAGCTTTACCCGCTCCTTTAAATGGTGAGCACATCATAAATAAT-------- 52

Ci17 TTTCCCCGAAGCTTTACCCGCTCCTTTAAATGGTGAGCACATCATAAATAAT-------- 52

********************************************* *****

WT --------CATGTTGTCAA-ACTTCCCTAAAAGTTATTAAATTGATTATTCAATTACACC 103

Ci11 --------CATGTTGTCAA-ACTTCCCTAAAAGTTATTAAATTGATTATTCAATTACACC 103

Ci12 --------CATGTTGTCAA-ACTTCCCTAAAAGTTATTAAATTGATTATTCAATTACACC 103

Ci13 TCTGTAATCCTGTTGTCAAACTTCCCTAATAAGTTATTAAATTGATTATTCAATTACACC 120

Ci14 TCTGTAATCCTGTTGTCAAACTTCCCTAATAAGTTATTAAATTGATTATTCAATTACACC 120

Ci15 TCTGTAATCCTGTTGTCAAACTTCCCTAATAAGTTATTAAATTGATTATTCAATTACACC 120

Ci16 --------CATGTTGTCAA-ACTTCCCTAAAAGTTATTAAATTGATTATTCAATTACACC 103

Ci17 --------CATGTTGTCAA-ACTTCCCTAAAAGTTATTAAATTGATTATTCAATTACACC 103

* ********* * ** * ******************************

WT TATGGGACTTTACTAACCTTAAAAGAGCAGTAAGGTTCATTACTTCTTCATCGGACCTTT 163

Ci11 TATGGGACTTTACTAACCTTAAAAGAGCAGTAAGGTTCATTACTTTTTCATCGGACCTTT 163

Ci12 TATGGGACTTTACTAACCTTAAAAGAGCAGTAAGGTTCATTACTTCTTCATCGGACCTTT 163

Ci13 TTTGGGACTTTACTAACCTTAAAAGAGCATTAAGGTTCATTACTTCTTCATCGGACCTTT 180

Ci14 TTTGGGACTTTACTAACCTTAAAAGAGCATTAAGGTTCATTACTTCTTCATCGGACCTTT 180

Ci15 TTTGGGACTTTACTAACCTTAAAAGAGCATTAAGGTTCATTACTTCTTCATCGGACCTTT 180

Ci16 TATGGGACTTTACTAACCTTAAAAGAGCAGTAAGGTTCATTACTTCTTCATCGGACCTTT 163

Ci17 TATGGGACTTTACTAACCTTAAAAGAGCAGTAAGGTTCATTACTTCTTCATCGGACCTTT 163

* *************************** *************** **************

WT TGTGTGCAGCTAAAGTGTTAACTGCTTTGATATAATTTGCAGGAGTTTTGGCCATCCTAA 223

Ci11 TGTGTGCAGCTAAAGTGTTAACTGCTTTGATATAATTTGCAGGAGTTTTGGCCATCCTAA 223

Ci12 TGTGTGCAGCTAAAGTGTTAACTGCTTTGATATAATTTGCAGGAGTTTTGGCCATCCTAA 223

Ci13 TGTGTGCAGCTAAAGTGTTAACTGCTTTGATATAATTTGCAGGAGTTTTGGCCATCCTAA 240

Ci14 TGTGTGCAGCTAAAGTGTTAACTGCTTTGATATAATTTGCAGGAGTTTTGGCCATCCTAA 240

Ci15 TGTGTGCAGCTAAAGTGTTAACTGCTTTGATATAATTTGCAGGAGTTTTGGCCATCCTAA 240

Ci16 TGTGTGCAGCTAAAGTGTTAACTGCTTTGATATAATTTGCAGGAGTTTTGGCCATCCTAA 223

Ci17 TGTGTGCAGCTAAAGTGTTAACTGCTTTGATATAATTTGCAGGAGTTTTGGCCATCCTAA 223

************************************************************

WT AGAACAATGAAATGCTTACATGGCCAGAGAAAGTCAAATTTGCAATTGGACTCTTGCCAG 283

Ci11 AGAACAATGAAATGCTTACATGGCCAAAGAAAGTCAAATTTGCAATTGGACTCTTGCCAG 283

Ci12 AGAACAATGAAATGCTTACATGGCCAGAGAAAGTCAAATTTGCAATTGGACTCTTGCCAG 283

Ci13 AGAACAATGAAATGCTTACATGGCCAGAGAAAGTCAAATTTGCAACTGGACTCTTGCCAG 300

Ci14 AGAACAATGAAATGCTTACATGGCCAGAGAAAGTCAAATTTGCAATTGGACTCTTGCCAG 300

Ci15 AGAACAATGAAATGCTTACATGGCCAGAGAAAGTCAAATTTGCAATTGGACTCTTGCCAG 300

Ci16 AGAACAATGAAATGCTTACATGGCCAGAGAAAGTCAAATTTGCAATTGGACTCTTGCCAG 283

Ci17 AGAACAATGAAATGCTTACATGGCCAGAGAAAGTCAAATTTGCAATTGGACTCTTGCCAG 283

************************** ****************** **************

WT CAATGCTTGGAGGGCAATCTTATGTTGGGGCTCAAGATGGGATAAGTGTCAAGGACTGGA 343

Ci11 CA-TGCTTGGAGGGCAATCTTATGTTGAAGCTCAAGACGGGATAAGTGTTAAGGACTGGA 342

Ci12 ---TGCTTGGAGGGCAATCTTATGTTGAAGCTCAAGACGGGATAAGTGTTAAGGACTGGA 340

Ci13 CAATGCTTGGAGGGCAATCTTATGTTGAGGCTCAAGATGGGATAAGTGTCAAGGACTGGA 360

Ci14 CA-TGCTTGGAGGGCAATCTTATGTTGAAGCTCAAGACGGGATAAGTGTTAAGGACTGGA 359

Ci15 CAATGCTTGGAGGGCAATCTTATGTTGAAGCTCAAGACGGGATAAGTGTTAAGGACTGGA 360

Ci16 CAATGCTTGGAGGGCAATCTTATGTTGAAGCTCAAGACGGGATAAGTGTTAAGGACTGGA 343

Ci17 CA-TGCTTGGAGGGCAATCTTATGTTGAAGCTCAAGACGGGATAAGTGTTAAGGACTGGA 342

************************ ******** *********** **********

WT TGAGAAAGCAAGTGCGTAAACAGTTATGTTACTTTTTAAGCATAAACCTGGCTGTATAGT 403

Ci11 TGAGAAAGCAAGTGCGTAATTAGTTATGTTACTTTTTAAGCATAAACCTGGCTGTATAGT 402

Ci12 TGAGAAAGCAAGTGCGTAATTAGTTATGTTACTTTTTAAGCATAAACCTGGCTGTATTGT 400

Ci13 TGAGAAAGCAAGTGCGTAATCAGTTATGTTACTTTTTAAGCATAAACCTGGCTGTATAGT 420

Ci14 TGAGAAAGCAAGTGCGTAATTAGTTATGTTACTTTTTAAGCATAAACCTGGCTGTATAGT 419

Ci15 TGAGAAAGCAAGTGCGTAATTAGTTATGTTACTTTTTAAGCATAAACCTGGCTGTATTGT 420

Ci16 TGAGAAAGCAAGTGCGTAATTAGTTATGTTACTTTTTAAGCATAAACCTGGCTGTATTGT 403

Ci17 TGAGAAAGCAAGTGCGTAATTAGTTATGTTACTTTTTAAGCATAAACCTGGCTGTATAGT 402

******************* ************************************ **

WT TCACAGTTCACACACAAATTGTTCATTTGAGGTGTTAGATTGTCTACCTGCCTTTCTTTT 463

Ci11 TCACAGTTCACAC--ACATTGTTCATTTGAGGTGTTAGATTGTCTACCTGCCTTTCTTTT 460

Ci12 TCACAGTTCACACACAAATTGTTCATTTGAGGTGTTAGATTGTCTACCTGCCTTTCTTTT 460

Ci13 TCACAGTTCACACACAAATTGTTCATTTGAGGTGTTAGATTGTCTACCTGCCTTTCTTTT 480

Ci14 TCACAGTTCACAC--ACATTGTTCATTTGAGGTGTTAGATTGTCTACCTGCCTTTCTTTT 477

Ci15 TCACAGTTCACACACAAATTGTTCATTTGAGGTGTTAGATTGTCTACCTGCCTTTCTTTT 480

Ci16 TCACAGTTCACACACAAATTGTTCATTTGAGGTGTTAGATTGTCTACCTGCCTTTCTTTT 463

Ci17 TCACAGTTCACAC--ACATTGTTCATTTGAGGTGTTAGATTGTCTACCTGCCTTTCTTTT 460

************* * *******************************************

WT GCTAACAGCATACTTCTAGCAAATCTCAGCAGCAGCAATTTGTTTTGCAGGGTGTG-CCG 522

Ci11 GCTAGCAGCATACATCTAGCAAATCTTAGCCGCAGCAATTTGTTTTGCGGGGGTGTGCCG 520

Ci12 GCTAGCCGCATACATCTAGCAAATCTTAGCCGCAGCAATTTGTTTTGCAGGGTG-TGCCG 519

Ci13 GCTAACAGCATACTTCTAGCAAATCTCAGCAGCAGCAATTTGTTTTGCAGGGTGTGCC-G 539

Ci14 GCTAGCAGCATACATCTAGCAAATCTTAGCCGCAGCAATTTGTTAAAACGGGGTGTGCCG 537

Ci15 GCTAGCAGCATACATCTAGCAAATCTTAGCCGCAGCAATTTGTTTTGCAGGGTGTGCC-G 539

Ci16 GCTAGCCGCATACATCTAGCAAATCTTAGCCGCAGCAATTTGTTTTGCAGGGTG-TGCCG 522

Ci17 GCTAGCCGCATACATCTAGCAAATCTTAGCCGCAGCAATTTGTTTTGCAGGGTG-TGCCG 519

**** * ****** ************ *** ************* *** * *

WT GATAGGGTGACAGAT 537

Ci11 GATAGGGTGACAGAT 535

Ci12 GATAGGGTGACAGAT 534

Ci13 GATAGGGTGACAGAT 554

Ci14 GATAGGGTGACAGAT 552

Ci15 GATAGGGTGACAGAT 554

Ci16 GATAGGGTGACAGAT 537

Ci17 GATAGGGTGACAGAT 534

***************

**Fig. S11** Multiple sequence alignment using Clustal Omega of *PDS* fragments cloned from the chimeric mutant plant PDS.9m. WT, DNA sequence of the D1 clone isolated from non-transformed ‘Désirée’. The 20-bp region corresponding to the sgRNA is in red and blue colours. The *Mly*I recognition site is in blue. Differences compared to WT are highlighted in green.

**PDS.10m MOSAIC plant „patchy”**

Ci16 TTTCCCCGAAGCTTTACCCGCTCCTTTAAATGGTGAGCACATCATAAATAATCATGTTGT 60

Ci22 TTTCCCCGAAGCTTTACCCGCTCCTTTAAATGGTGAGCACATCATAAATAATCATGTTGT 60

Ci21 TTTCCCCGAAGCTTTACCCGCTCCTTTAAATGGTGAGCACATCATAAATAATCATGTTGT 60

WT TTTCCCCGAAGCTTTACCCGCTCCTTTAAATGGTGAGCACATCATAAATAATCATGTTGT 60

Ci23 TTTCCCCGAAGCTTTACCCGCTCCTTTAAATGGTGAGCACATCATAAATAATCATGTTGT 60

************************************************************

Ci16 CAAACTTCCCTAAAAGTTATTAAATTGATTATTCAATTACACCTATGGGACTTTACTAAC 120

Ci22 CAAACTTCCCTAAAAGTTATTAAATTGATTATTCAATTACACCTATGGGACTTTACTAAC 120

Ci21 CAAACTTCCCTAAAAGTTATTAAATTGATTATTCAATTACACCTATGGGACTTTACTAAC 120

WT CAAACTTCCCTAAAAGTTATTAAATTGATTATTCAATTACACCTATGGGACTTTACTAAC 120

Ci23 CAAACTCCCCTAAAAGTTATTAAATTGATTATTCAATTACACCTATGGGACTTTACTAAC 120

****** *****************************************************

Ci16 CTTAAAAGAGCAGTAAGGTTCATTACTTCTTCATCGGACCTTTTGTGTGCAGCTAAAGTG 180

Ci22 CTTAAAAGAGCAGTAAGGTTCATTACTTCTTCATCGGACCTTTTGTGTGCAGCTAAAGTG 180

Ci21 CTTAAAAGAGCAGTAAGGTTCATTACTTCTTCATCGGACCTTTTGTGTGCAGCTAAAGTG 180

WT CTTAAAAGAGCAGTAAGGTTCATTACTTCTTCATCGGACCTTTTGTGTGCAGCTAAAGTG 180

Ci23 CTTAAAAGAGCATTAAGGTTCATTACTTCTTCATCGGACCTTTTGTGTGCAGCTAAAGTG 180

************ ***********************************************

Ci16 TTAACTGCTTCGATATAATTTGCAGGTTACTTTTTAAGCATAAACCTGGCTGTATTGTTC 240

Ci22 TTAACTGCTTTGATATAATTTGCAGGAGTTTTGGCCATCCTAAAGAACAATGAAATGCTT 240

Ci21 TTAACTGCTTTGATATAATTTGCAGGAGTTTTGGCCATCCTAAAGAACAATGAAATGCTT 240

WT TTAACTGCTTTGATATAATTTGCAGGAGTTTTGGCCATCCTAAAGAACAATGAAATGCTT 240

Ci23 TTAACTGCTTTGATATAATTTGCAGGAGTTTTGGCCATCCTAAAGAACAATGAAATGCTT 240

********** *************** ** * * **** ** * ** *

Ci16 ACA--------------------------------------------------------- 292

Ci22 ACATGGCCAGAGAAAGTCAAATTTGCAATTGGACTCTTGCCAGCAATGCTTGGAGGGCAA 300

Ci21 ACATGGCCAGAGAAAGTCAAATTTGCAATTGGACTCTTGCCAGCAATGCTTGGAGGGCAA 300

WT ACATGGCCAGAGAAAGTCAAATTTGCAATTGGACTCTTGCCAGCAATGCTTGGAGGGCAA 300

Ci23 ACATGGCCAGAGAAAGTCAAATTTGCAATTGGACTCTTGCCAGCAATGCTTGGAGGGCAA 300

*** Ci16 ------------------------------------------------------------ 315

Ci22 TCTTATGTTGAAGCTCAAGACGGGATAAGTGTTAAGGACTGGATGAGAAAGCAAGTGCGT 360

Ci21 TCTTATGTTGAAGCTCAAGACGGGATAAGTGTTAAGGACTGGATGAGAAAGCAAGTGCGT 360

WT TCTTATGTTGGGGCTCAAGATGGGATAAGTGTCAAGGACTGGATGAGAAAGCAAGTGCGT 360

Ci23 TCTTATGTTGAGGCTCAAGATGGGATAAGTGTCAAGGACTGGATGAGAAAGCAAGTGCGT 360

Ci16 ------------------------------------------------GTTCACACACAA 315

Ci22 AATTAGTTATGTTACTTTTTAAGCATAAACCTGGCTGTATTGTTCACAGTTCACACACAA 420

Ci21 AATTAGTTATGTTACTTTTTAAGCATAAACCTGGCTGTATTGTTCACAGTTCACACACAA 420

WT AAACAGTTATGTTACTTTTTAAGCATAAACCTGGCTGTATAGTTCACAGTTCACACACAA 420

Ci23 AATCAGTTATGTTACTTTTTAAGCATAAACCTGGCTGTATAGTTCACAGTTCACACACAA 420

************

Ci16 ATTGTTCATTTGAGGTGTTAGATTGTCTACCTGCCTTTCTTTTGCTAGCAGCATACATCT 316

Ci22 ATTGTTCATTTGAGGTGTTAGATTGTCTACCTGCCTTTCTTTTGCTAGCCGCATACATCC 480

Ci21 ATTGTTCATTTGAGGTGTTAGATTGTCTACCTGCCTTTCTTTTGCTAACAGCATACTTCT 480

WT ATTGTTCATTTGAGGTGTTAGATTGTCTACCTGCCTTTCTTTTGCTAACAGCATACTTCT 480

Ci23 ATTGTTCATTTGAGGTGTTAGATTGTCTACCTGCCTTCCTTTTGCTAACAGCATACTTCT 480

************************************* *********************

Ci16 AGCAAATCTTAGCCGCAGCAATTTGTTTTGCNNGGTGTGCCGGATAGGGTGACAGAT 373

Ci22 AGCAAATCTTAGCCGCAGCAATTTGTTTTGCAGGGTGTGCCGGATAGGGTGACAGAT 537

Ci21 AGCAAATCTCAGCAGCAGCAATTTGTTTTGCAGGGTGTGCCGGATAGGGTGACAGAT 537

WT AGCAAATCTCAGCAGCAGCAATTTGTTTTGCAGGGTGTGCCGGATAGGGTGACAGAT 537

Ci23 AGCAAATCTCAGCAGCAGCAATTTGTTTTGCAGGGTGTGCCGGATAGGGTGACAGAT 537

********* *** ***************** ************************

**Fig. S12** Multiple sequence alignment using Clustal Omega of *PDS* fragments cloned from the chimeric mutant plant PDS.10m. WT, DNA sequence of the D1 clone isolated from non-transformed ‘Désirée’. The 20-bp region corresponding to the sgRNA is in red and blue colours. The *Mly*I recognition site is in blue. Differences compared to WT are highlighted in green.

**PDS.10g GREEN plant**

10.3 TTTCCCCGAAGCTTTACCCGCTCCTCTAAATGGTGAGCACATCATAGATAATCATGTTGT 60

10.12 TTTCCCCGAAGCTTTACCCGCTCCTTTAAATGGTGAGCACATCATAAATAATCATGTTGT 60

10.52 TTTCCCCGAAGCTTTACCCGCTCCCTTAAATGGTGAGCACATCATAAATAATCATGTTGT 60

WT TTTCCCCGAAGCTTTACCCGCTCCTTTAAATGGTGAGCACATCATAAATAATCATGTTGT 60

10.1 TTTCCCCGAAGCTTTACCCGCTCCTTTAAATGGTGAGCACATCATAAATAATCATGTTGT 60

************************ ******************** *************

10.3 CAAACTTCCCTAAAAGTTATTAAATTGATTATTCAGTTACACCTATGGGACCTTACTAAC 120

10.12 CAAACTTCCCTAAGAGTCATTAAATTGATTATTCAATTACACCTATGGGACTTTACTAAC 120

10.52 CAAACTTCCCTAAAAGTTATTAAATTGATTATTCAATTACACCTATGGGGCTTTACTAAC 120

WT CAAACTTCCCTAAAAGTTATTAAATTGATTATTCAATTACACCTATGGGACTTTACTAAC 120

10.1 CAAACTTCCCTAAAAGTTATTAAATTGATTATTCAATTACACCTATGGGACTTTACTAAC 120

************* *** ***************** ************* * ********

10.3 CTTAAAAGAGCAGTAAGGTTCATTACTTTTTCATCGGACCTTTTGTGTGCAGCTAAAGTG 180

10.12 CTTAAAAGAGCAGTAAGGTTCATTACTTCTTCATCGGACCTTTTGTGTGCAGCTAAAGTG 180

10.52 CTTAAAAGAGCAGTAAGGTTCATTACTTCTTCATCGGACCTTTTGTGTGCAGCTAAAGTG 180

WT CTTAAAAGAGCAGTAAGGTTCATTACTTCTTCATCGGACCTTTTGTGTGCAGCTAAAGTG 180

10.1 CTTAAAAGAGCAGTAAGGTTCATTACTTCTTCATCGGACCTTTTGTGTGCAGCTAAAGTG 180

**************************** *******************************

10.3 TTAACTGCTTTGATATAATTTGCAGGAGTTTTGGCCATCCTAAGGAACAATGAAATGCTT 240

10.12 TTAACTGCTTTGATATAATTTGCAGGAGTTTTGGCCATCCTAAAGAACAATGAAATGCTT 240

10.52 TTAACTGCTTTGATATAATTTGCAGGAGTTTTGGCCATCCTAAAGAACAATGAAATGCTT 240

WT TTAACTGCTTTGATATAATTTGCAGGAGTTTTGGCCATCCTAAAGAACAATGAAATGCTT 240

10.1 TTAACTGCTTTGATATAATTTGCAGGAGTTTTGGCCATCCTAAAGAACAATGAAATGCTT 240

******************************************* ****************

10.3 ACATGGCCAGAGAAAGTCAAATTTGCAATTGGACTCTTGCCAGCAATGCTTGGAGGGCAA 300

10.12 ACATGGCCAGAGAAAGTCAAATTTG-AATTGGACTCTTGCCAG---TGCTTGGAGGGCAA 296

10.52 ACATGGCCAGAGAAAGTCAAATTTGCAATTGGACTCTTGCCAGCAATGCTTGGAGGGCAA 300

WT ACATGGCCAGAGAAAGTCAAATTTGCAATTGGACTCTTGCCAGCAATGCTTGGAGGGCAA 300

10.1 ACATGGCCAGAGAAAGTCAAATTTGCAATTGGACTCTTGCCAGCAATGCTTGGAGGGCAA 300

************************* ***************** **************

10.3 TCTTATGTTGAAGCTCAAGACGGGATAAGTGTTAAGGACTGGATGAGAAAGCAAGTGCGT 360

10.12 TCTTATGTTGAAGCTCAAGACGGGATAAGTGTTAAGGACTGGATGAGAAAGCAAGTGCGT 356

10.52 TCTTATGTTGAAGCTCAAGACGGGATAAGTGTTAAGGACTGGATGAGAAAGCAAGTGCGT 360

WT TCTTATGTTGGGGCTCAAGATGGGATAAGTGTCAAGGACTGGATGAGAAAGCAAGTGCGT 360

10.1 TCTTATGTTGAGGCTCAAGATGGGATAAGTGCCAAGGACTGGATGAGAAAGCAAGTGCGT 360

********** ******** ********** ***************************

10.3 AATTAGTTATGTTACTTTTTAAGCATAAACCTGGCTGTATAGTTCACAGTTCACAC--AA 418

10.12 AATTAGTTATGTTACTTTTTAAGCATAAACCTGGCTGTATTGTTCACAGTTCACACACAA 416

10.52 AATTAGTTATGTTACTTTTTAAGCATAAACCTGGCTGTATTGTTCACAGTTCACACACAA 420

WT AAACAGTTATGTTACTTTTTAAGCATAAACCTGGCTGTATAGTTCACAGTTCACACACAA 420

10.1 AATCAGTTATGTTACTTTTTAAGCATAAACCTGGCTGTATAGTTCACAGTTCACACACAA 420

** ************************************ *****************

10.3 ATTGTTCATTTGAGGTGCTAGATTGTCTACCTGCCTTTCTTTTGCTAGCAGCATACATCT 478

10.12 ATTGTTCATTTGAGGTGTTAGATTGCCTACCTGCCTTTCTTTTGCTAGCCGCATACATCT 476

10.52 ATTGTTCACTTGAGGTGTTAGATTGTCTACCTGCCTTTCTTTTGCTAGCCGCATACATCT 480

WT ATTGTTCATTTGAGGTGTTAGATTGTCTACCTGCCTTTCTTTTGCTAACAGCATACTTCT 480

10.1 ATTGTTCATTTGAGGTGTTAGATTGTCTACCTGCCTTTCTTTTGCTAACAGCATACTTCT 480

******** ******** ******* ********************* * ****** ***

10.3 AGCAAATCTTAGCCGCAGCAATTTGTTTTGCAGGGTGTGCCGGATAGGGTGACAGAT 535

10.12 AGCAAATCTTAGCCGCAGCAATTTGTTTTGCAGGGTGTGCCGGATAGGGTGACAGAT 533

10.52 AGCAAATCTTAGCCGCAGCAATTTGTTTTGCAGGGTGTGCCGGATAGGGTGACAGAT 537

WT AGCAAATCTCAGCAGCAGCAATTTGTTTTGCAGGGTGTGCCGGATAGGGTGACAGAT 537

10.1 AGCAAATCTCAGCAGCAGCAATTTGTTTTGCAGGGTGTGCCGGATAGGGTGACAGAT 537

********* *** *******************************************

**Fig. S13** Multiple sequence alignment using Clustal Omega of *PDS* fragments cloned from the green mutant plant PDS.10g. WT, DNA sequence of the D1 clone isolated from non-transformed ‘Désirée’. The 20-bp region corresponding to the sgRNA is in red and blue colours. The *Mly*I recognition site is in blue. Differences compared to WT are highlighted in green.

**PDS.1g GREEN plant**

WT TTTCCCCGAAGCTTTACCCGCTCCTTTAAATGGTGAGCACATCATAAATAATCATGTTGT 60

1.3 TTTCCCCGAAGCTTTACCCGCTCCTTTAAATGGTGAGCACATCATAAATAATCATGTTGT 60

1.1 TTTCCCCGAAGCTTTACCCGCTCCTTTAAATGGTGAGCACATCATAAATAATCATGTTGT 60

1.9 TTTCCCCGAAGCTTTACCCGCTCCTTTAAATGGTGAGCACATCATAAATAATCATGTTGT 60

1.11 TTTCCCCGAAGCTTTACCCGCTCCTTTAAATGGTGAGCACATCATAAATAATCATGTTGT 60

1.7 TTTCCCCGAAGCTTTACCCGCTCCTTTAAATGGTGAGCACATCATAAATAATCATGTTGT 60

1.4 TTTCCCCGAAGCTTTACCCGCTCCTTTAAATGGTGAGCACATCATAAATAATCATGTTGT 60

1.5 TTTCCCCGAAGCTTTACCCGCTCCTTTAAATGGTGAGCACATCATAAATAATCATGTTGT 60

************************************************************

WT CAAACTTCCCTAA-AAGTTATTAAATTGATTATTCAATTACACCTATGGGACTTTACTAA 119

1.3 CAAACTTCCCTAA-AAGTTATTAAATTGATTATTCAATTACACCTTTGGGACTTTACTAA 119

1.1 CAAACTTCCCTAA-AAGTTATTAAATTGATTATTCAATTACACCTATGGGACTTTACTAA 119

1.9 CAAACTTCCCTAATAAGTTATTAAATTGATTATTCAATTACACCTTTGGGACTTTACTAA 120

1.11 CAAACTTCCCTAA-AAGTTATTAAATTGATTATTCAATTACACCTATGGGACTTTACTAA 119

1.7 CAAACTTCCCTAA-AAGTTATTAAATTGATTATTCAATTACACCTATGGGACTTTACTAA 119

1.4 CAAACTTCCCTAA-AAGTTATTAAATTGATTATTCAATTACACCTATGGGACTTTACTAA 119

1.5 CAAACTTCCCTAA-AAGTTATTAAATTGATTATTCAATTACACCTATGGGACTTTACTAA 119

************* ******************************* **************

WT CCTTAAAAGAGCAGTAAGGTTCATTACTTCTTCATCGGACCTTTTGTGTGCAGCTAAAGT 179

1.3 CCTTAAAAGAGCATTAAGGTTCATTACTTCTTCATCGGACCTTTTGTGTGCAGCTAAAGT 179

1.1 CCTTAAAAGAGCAGTAAGGTTCATTACTTCTTCATCGGACCTTTTGTGTGCAGCTAAAGT 179

1.9 CCTTAAAAGAGCAGTAAGGTTCATTACTTCTTCATCGGACCTTTTGTGTGCAGCTAAAGT 180

1.11 CCTTAAAAGAGCAGTAAGGTTCATTACTTTTTCATCGGACCTTTTGTGTGCAGCTAAAGT 179

1.7 CCTTAAAAGAGCAGTAAGGTTCATTACTTTTTCATCGGACCTTTTGTGTGCAGCTAAAGT 179

1.4 CCTTAAAAGAGCAGTAAGGTTCATTACTTCTTCATCGGACCTTTTGTGTGCAGCTAAAGT 179

1.5 CCTTAAAAGAGCAGTAAGGTTCATTACTTCTTCATCGGACCTTTTGTGTGCAGCTAAAGT 179

************* *************** ******************************

WT GTTAACTGCTTTGATATAATTTGCAGGAGTTTTGGCCATCCTAAAGAACAATGAAATGCT 239

1.3 GTTAACTGCTTTGATATAATTTGCAGGAGTTTTGGCCATCCTAAAGAACAATGAAATGCT 239

1.1 GTTAACTGCTTTGATATAATTTGCAGGAGTTTTGGCCATCCTAAAGAACAATGAAATGCT 239

1.9 GTTAACTGCTTTGATATAATTTGCAGGAGTTTTGGCCATCCTAAAGAACAATGAAATGCT 240

1.11 GTTAACTGCTTTGATATAATTTGCAGGAGTTTTGGCCATCCTAAAGAACAATGAAATGCT 239

1.7 GTTAACTGCTTTGATATAATTTGCAGGAGTTTTGGCCATCCTAAAGAACAATGAAATGCT 239

1.4 GTTAACTGCTTTGATATAATTTGCAGGAGTTTTGGCCATCCTAAAGAACAATGAAATGCT 239

1.5 GTTAACTGCTTTGATATAATTTGCAGGAGTTTTGGCCATCCTAAAGAACAATGAAATGCT 239

************************************************************

WT TACATGGCCAGAGAAAGTCAAATTTGCAATTGGACTCTTGCCAGCAATGCTTGGAGGGCA 299

1.3 TACATGGCCAGAGAAAGTCAAATTTGCAATTGGACTCTTGCCAGCAATGCTTGGAGGGCA 299

1.1 TACATGGCCAGAGAAAGTCA**G**ATTTGCAATTG--CTCTTGCCAGCAATGCTTGGAGGGCA 299

1.9 TACATGGCCAGAGAAAGTCAAATTTGCAATTGGACTCTTGCCAGCAATGCTTGGAGGGCA 300

1.11 TACATGGCCAGAGAAAGTCAAATTTGCAATTGGACTCTTGCCAGCAATGCTTGGAGGGCA 299

1.7 TACATGGCCAGAGAAAGTCAAATTTGCAATTGGACTCTTGCCAGCAATGCTTGGAGGGCA 299

1.4 TACATGGCCAGAGAAAGTCAAATTTGCAATTGGACTCTTGCCAGCAATGCTTGGAGGGCA 299

1.5 TACATGGCCAGAGAAAGTCAAATTTGCAATTGGACTCTTGCCAGCAATGCTTGGAGGGCA 299

******************** ***************************************

WT ATCTTATGTTGGGGCTCAAGATGGGATAAGTGTCAAGGACTGGATGAGAAAGCAAGTGCG 359

1.3 ATCTTATGTTGAGGCTCAAGATGGGATAAGTGTCAAGGACTGGATGAGAAAGCAAGTGCG 359

1.1 ATCTTATGTTGAAGCTCAAGACGGGATAAGTGTCAAGGACTGGATGAGAAAGCAAGTGCG 359

1.9 ATCTTATGTTGAAGCTCAAGACGGGATAAGTGTTAAGGACTGGATGAGAAAGCAAGTGCG 360

1.11 ATCTTATGTTGAAGCTCAAGACGGGATAAGTGTTAAGGACTGGATGAGAAAGCAAGTGCG 359

1.7 ATCTTATGTTGAAGCTCAAGACGGGATAAGTGTTAAGGACTGGATGAGAAAGCAAGTGCG 359

1.4 ATCTTATGTTGAAGCTCAAGACGGGATAAGTGTTAAGGACTGGATGAGAAAGCAAGTGCG 359

1.5 ATCTTATGTTGAAGCTCAAGACGGGATAAGTGTTAAGGACTGGATGAGAAAGCAAGTGCG 359

*********** ******** *********** **************************

WT TAAACAGTTATGTTACTTTTTAAGCATAAACCTGGCTGTATAGTTCACAGTTCACACACA 419

1.3 TAATCAGTTATGTTACTTTTTAAGCATAAACCTGGCTGTATAGTTCACAGTTCACACACA 419

1.1 TAATCAGTTATGTTACTTTTTAAGCATAAACCTGGCTGTATAGTTCACAGTTCACACACA 419

1.9 TAATTAGTTATGTTACTTTTTAAGCATAAACCTGGCTGTATTGTTCACAGTTCACACACA 420

1.11 TAATTAGTTATGTTACTTTTTAAGCATAAACCTGGCTGTATTGTTCACAGTTCACACACA 419

1.7 TAATTAGTTATGTTACTTTTTAAGCATAAACCTGGCTGTATTGTTCACAGTTCACACACA 419

1.4 TAATTAGTTATGTTACTTTTTAAGCATAAACCTGGCTGTATTGTTCACAGTTCACACACA 419

1.5 TAATTAGTTATGTTACTTTTTAAGCATAAACCTGGCTGTATTGTTCACAGTTCACACACA 419

*** ************************************ ******************

WT AATTGTTCATTTGAGGTGTTAGATTGTCTACCTGCCTTTCTTTTGCTAACAGCATACTTC 479

1.3 AATTGTTCATTTGAGGTGTTAGATTGTCTACCTGCCTTTCTTTTGCTAACAGCATACTTC 479

1.1 AATTGTTCATTTGAGGTGTTAGATTGTCTACCTGCCTTTCTTTTGCTAACAGCATACTTC 479

1.9 AATTGTTCATTTGAGGTGTTAGATTGTCTACCTGCCTTTCTTTTGCTAGCCGCATACATC 480

1.11 AATTGTTCATTTGAGGTGTTAGATTGTCTACCTGCCTTTCTTTTGCTAGCCGCATACATC 479

1.7 AATTGTTCATTTGAGGTGTTAGATTGTCTACCTGCCTTTCTTTTGCTAGCCGCATACATC 479

1.4 AATTGTTCATTTGAGGTGTTAGATTGTCTACCTGCCTTTCTTTTGCTAGCCGCATACATC 479

1.5 AATTGTTCATTTGAGGTGTTAGATTGTCTACCTGCCTTTCTTTTGCTAGCCGCATACATC 479

************************************************ * ****** **

WT TAGCAAATCTCAGCAGCAGCAATTTGTTTTGCAGGGTGTGCCGGATAGGGTGACAGAT 537

1.3 TAGCAAATCTCAGCAGCAGCAATTTGTTTTGCAGGGTGTGCCGGATAGGGTGACAGAT 537

1.1 TAGCAAATCTCAGCAGCAGCAATTTGTTTTGCAGGGTGTGCCGGATAGGGTGACAGAT 537

1.9 TAGCAAATCTTAGCCGCAGCAATTTGTTTTGCAGGGTGTGCCGGATAGGGTGACAGAT 538

1.11 TAGCAAATCTTAGCCGCAGCAATTTGTTTTGCAGGGTGTGCCGGATAGGGTGACAGAT 537

1.7 TAGCAAATCTTAGCCGCAGCAATTTGTTTTGCAGGGTGTGCCGGATAGGGTGACAGAT 537

1.4 TAGCAAATCTTAGCCGCAGCAATTTGTTTTGCAGGGTGTGCCGGATAGGGTGACAGAT 537

1.5 TAGCAAATCTTAGCCGCAGCAATTTGTTTTGCAGGGTGTGCCGGATAGGGTGACAGAT 537

********** *** *******************************************

**Fig. S14** Multiple sequence alignment using Clustal Omega of *PDS* fragments cloned from the green mutant plant PDS.1g. WT, DNA sequence of the D1 clone isolated from non-transformed ‘Désirée’. The 20-bp region corresponding to the sgRNA is in red and blue colours. The *Mly*I recognition site is in blue. Differences compared to WT are highlighted in green.

**PDS.33g GREEN plant**

33.8 TTTCCCCGAA-CTTTA-CCCGCTCCTTTTTATGGTGAGCACATCATAAATAATCATGTTG 58

33.6 TTTCCCCGAAGCTTTA-CCCGCTCCTTTAAATGGTGAGCACATCATAAATAATCATGTTG 59

WT TTTCCCCGAAGCTTTA-CCCGCTCCTTTAAATGGTGAGCACATCATAAATAATCATGTTG 59

33.5 TTTCCCCGAAGCTTTA-CCCGCTCCTTTAAATGGTGAGCACATCATAAATAATCATGTTG 59

33.12 TTTCCCCGAAGCTTTA-CCCGCTCCTTTAAATGGTGAGCACATCATAAATAATCATGTTG 59

33.3 TTTCCCCGAAGCTTTACCCG-CTCCTTTAAATGGTGAGCACATCATAAATAATCATGTTG 59

33.7 TTTCCCCGAAGCTTTACCCG-CTCCTTNAATANGTGAGCACATCATAAATAATCATGTTG 60

33.10 TTTCCCCGAAGCTTTACCCG--TCCTTTAAATGGTGAGCACATCATAAATAATCATGTTG 59

33.1 TTTCCCCGAAGCTTTACCCG-CTCCTTTAAATGGTGAGCACATCATAAATAATCATGTTG 59

33.2 TTTCCCCGAAGCTTTACCCG-CTCCTTTAAATGGTGAGCACATCATAAATAATCATGTTG 59

********** **** ** **** ***************************

33.8 TCAAACTTCCCTAAAAGTTATTAAATTGATTATTCAATTACACCTATGGGACTTTACTAA 118

33.6 TCAAACTTCCCTAAAAGTTATTAAATTGATTATTCAATTACACCTTTGGGACTTTACTAA 119

WT TCAAACTTCCCTAAAAGTTATTAAATTGATTATTCAATTACACCTATGGGACTTTACTAA 119

33.5 TCAAACTTCCCTAAAAGTTATTAAATTGATTATTCAATTACACCTATGGGACTTTACTAA 119

33.12 TCAAACTTCCCTAAAAGTTATTAAATTGATTATTCAATTACACCTATGGGACTTTACTAA 119

33.3 TCAAACTTCCCTAAAAGTTATTAAATTGATTATTCAATTACACCTATGGGACTTTACTAA 119

33.7 TCAAACTTCCCTAAAAGTTATTAAATTGATTATTCAATTACACCTATGGGACTTTACTAA 120

33.10 TCAAACTTCCCTAAAAGTTATTAAATTGATTATTCAATTACACCTATGGGACTTTACTAA 119

33.1 CCAAACTTCCCTAAAAGTTATTAAATTGATTATTCAATTACACCTATGGGACTTTACTAA 119

33.2 CCAAACTTCCCTAAAAGTTATTAAATTGATTATTCAATTACACCTATGGGACTTTACTAA 119

******************************************** **************

33.8 CCTTAAAAGAGCAGTAAGGTTCATTACTTCTTCATCGGACCTTTTGTGTGCAGCTAAAGT 178

33.6 CCTTAAAAGAGCATTAAGGTTCATTACTTCTTCATCGGACCTTTTGTGTGCAGCTAAAGT 179

WT CCTTAAAAGAGCAGTAAGGTTCATTACTTCTTCATCGGACCTTTTGTGTGCAGCTAAAGT 179

33.5 CCTTAAAAGAGCAGTAAGGTTCATTACTTCTTCATCGGACCTTTTGTGTGCAGCTAAAGT 179

33.12 CCTTAAAAGAGCATTAAGGTTCATTACTTCTTCATCGGACCTTTTGTGTGCAGCTAAAGT 179

33.3 CCTTAAAAGAGCAGTAAGGTTCATTACTTCTTCATCGGACCTTTTGTGTGCAGCTAAAGT 179

33.7 CCTTAAAAGAGCAATAAGGTTCATTACTTCTTCATCGGACCTTTTGTGTGCAGCTAAAGT 180

33.10 CCTTAAAAGAGCAGTAAGGTTCATTACTTCTTCATCGGACCTTTTGTGTGCAGCTAAAGT 179

33.1 CCTTAAAAGAGCAGTAAGGTTCATTACTTCTTCATCGGACCTTTTGTGTGCAGCTAAAGT 179

33.2 CCTTAAAAGAGCAGTAAGGTTCATTACTTCTTCATCGGACCTTTTGTGTGCAGCTAAAGT 179

************* **********************************************

33.8 GTTAACTGCTTTGATATAATTTGCAGGAGTTTTGGCCATCCTAAAGAACAATGAAATGCT 238

33.6 GTTAACTGCTTTGATATAATTTGCAGGAGTTTTGGCCATCCTAAAGAACAATGAAATGCT 239

WT GTTAACTGCTTTGATATAATTTGCAGGAGTTTTGGCCATCCTAAAGAACAATGAAATGCT 239

33.5 GTTAACTGCTTTGATATAATTTGCAGGAGTTTTGGCCATCCTAAAGAACAATGAAATGCT 239

33.12 GTTAACTGCTTTGATATAATTTGCAGGAGTTTTGGCCATCCTAAAGAACAATGAAATGCT 239

33.3 GTTAACTGCTTTGATATAATTTGCAGGAGTTTTGGCCATCCTAAAGAACAATGAAATGCT 239

33.7 GTTAACTGCTTTGATATAATTTGCAGGAGTTTTGGCCATCCTAAAGAACAATGAAATGCT 240

33.10 GTTAACTGCTTTGATATAATTTGCAGGAGTTTTGGCCATCCTAAAGAACAATGAAATGCT 239

33.1 GTTAACTGCTTTGATATAATTTGCAGGAGTTTTGGCCATCCTAAAGAACAATGAAATACT 239

33.2 GTTAACTGCTTTGATATAATTTGCAGGAGTTTTGGCCATCCTAAAGAACAATGAAATGCT 239

********************************************************* **

33.8 TACATGGCCAGAGAAAGTCAAATTTGCAATTGGA--CTTGCCA**A**CAATGCTTGGAGGGCA 298

33.6 TACATGGCCAGAGAAAGTCAAATTTGCAATTGGACTCTTGCCAGCAATGCTTGGAGGGCA 299

WT TACATGGCCAGAGAAAGTCAAATTTGCAATTGGACTCTTGCCAGCAATGCTTGGAGGGCA 299

33.5 TACATGGCCAGAGAAAGTCAAATTTGCAATTGGACTCTTGCCAGCAATGCTTGGAGGGCA 299

33.12 TACATGGCCAGAGAAAGTCAAATTTGCAATTGGACTCTTGCCAGCAATGCTTGGAGGGCA 299

33.3 TACATGGCCAGAGAAAGTCAAATTTGCAATTGGACTCTTGCCAGCAATGCTTGGAGGGCA 299

33.7 TACATGGCCAGAGAAAGTCAAATTTGCAATTGGACTCTTGCCAGCAATGCTTGGAGGGCA 300

33.10 TACATGGCCAGAGAAAGTCAAATTTGCAATTGGACTCTTGCCAGCAATGCTTGGAGGGCA 299

33.1 TACATGGCCAGAGAAAGTCAAATTTGCAATTGGACTCTTGCCAGCAATGCTTGGAGGGCA 299

33.2 TACATGGCCAGAGAAAGTCAAATTTGCAATTGGACTCTTGCCAGCAATGCTTGGAGGGCA 299

******************************************* ****************

33.8 ATCTTATGTTGAGGCTCAAGATGGGATAAGTGTCAAGGACTGGATGAGAAAGCAAGTGCG 358

33.6 ATCTTATGTTGAAGCTCAAGACGGGATAAGTGTTAAGGACTGGATGAGAAAGCAAGTGCG 359

WT ATCTTATGTTGGGGCTCAAGATGGGATAAGTGTCAAGGACTGGATGAGAAAGCAAGTGCG 359

33.5 ATCTTATGTTGAGGCTCAAGATGGGATAAGTGTCAAGGACTGGATGAAAAAGCAAGTGCG 359

33.12 ATCTTATGTTGAGGCTCAAGATGGGATAAGTGTCAAGGACTGGATGAGAAAGCAAGTGCG 359

33.3 ATCTTATGTTGAGGCTCAAGATGGGATAAGTGTCAAGGACTGGATGAGAAAGCAAGTGCG 359

33.7 ATCTTATGTTGAAGCTCGAGACGGGATAAGTGTTAAGGACTGGATGAGAAAGCAAGTGCG 360

33.10 ATCTTATGTTGAAGCTCAAGACGGGATAAGTGTTAAGGACTGGATGAGAAAGCAAGTGCG 359

33.1 ATCTTATGTTGAAGCTCAAGACGGGATAAGTGTTAAGGACTGGATGAGAAAGCAAGTGCG 359

33.2 ATCTTATGTTGAAGCTCAAGACGGGATAAGTGTTAAGGACTGGATGAGAAAGCAAGTGCG 359

*********** **** *** *********** ************* ************

33.8 TAATCAGTTATGTTACTTTTTAAGCATAAACCTGGCTGTATAGTTCACAGTTCACACACA 418

33.6 TAATCNGTTATGTTACTTTTTAAGCATAAACCTGGCTGTATAGTTCACAGTTCACACACA 419

WT TAAACAGTTATGTTACTTTTTAAGCATAAACCTGGCTGTATAGTTCACAGTTCACACACA 419

33.5 TAATCAGTTATGTTACTTTTTAAGCATAAACCTGGCTGTATAGTTCACAGTTCACACACA 419

33.12 TAATCAGTTATGTTACTTTTTAAGCATGAACCTGGCTGTATAGTTCACAGTTCACACACA 419

33.3 TAATCAGTTATGTTACTTTTTAAGCATAAACCTGGCTGTATAGTTCACAGTTCACACACA 419

33.7 TAATTAGTTATGTTACTTTTTAAGCATAAACCTGGCTGTATTGTTCACAGTTCACACACA 420

33.10 TAATTAGTTATGTTACTTTTTAAGCATAAACCTGGCTGTATTGTTCACAGTTCACACACA 419

33.1 TAATTAGTTATGTTACTTTTTAAGCATAAACCTGGCTGTATTGTTCACAGTTCACACACA 419

33.2 TAATTAGTTATGTTACTTTTTAAGCATAAACCTGGCTGTATTGTTCACAGTTCACACACA 419

*** ********************* ************* ******************

33.8 AATTGTTCATTTGAGGTGTTAGATTGTCTACCTGCCTTTCTTTTGCTAACAGCATACTTC 478

33.6 AATTGTTCATTTGAGGTGTTAGATTGTCTACCTGCCTTTCTTTTGCTAACAGCATACTTC 479

WT AATTGTTCATTTGAGGTGTTAGATTGTCTACCTGCCTTTCTTTTGCTAACAGCATACTTC 479

33.5 AATTGTTCATTTGAGGTGTTAGATTGTCTACCTGCCTTTCTTTTGCTAACAGCATACTTC 479

33.12 AATTGTTCATTTGAGGTGTTAGATTGTCTACCTGCCTTTCTTTTGCTAACAGCATACTTC 479

33.3 AATTGTTCATTTGAGGTGTTAGATTGTCTACCTGCCTTTCTTTTGCTAGCCGCATACATC 479

33.7 AATTGTTCATTTGAGGTGTTAGATTGTCTACCTGCCTTTCTTTTGCTAGCCGCATACATC 480

33.10 AATTGTTCATTTGAGGTGTTAGATTGTCTACCTGCCTTTCTTTTGCTAGCCGCATACATC 479

33.1 AATTGTTCATTTGAGGTGTTAGATTGTCTACCTGCCTTTCTTTTGCTAGCCGCATACATC 479

33.2 AATTGTTCATTTGAGGTGTTAGATTGTCTACCTGCCTTTCTTTTGCTAGCCGCATACATC 479

************************************************ * ****** **

33.8 TAGCAAATCTCAGCAGCAGCAATTTGTATTG-CAGGGTGTGCCGGATAGGGTGACAGAT 536

33.6 TAGCAAATCTCAGCAGCAGCAATTTGTTTTG-CAGGGTGTGCCGGATAGGGTGACAGAT 537

WT TAGCAAATCTCAGCAGCAGCAATTTGTTTTG-CAGGGTGTGCCGGATAGGGTGACAGAT 537

33.5 TAGCAAATCTCAGCAGCAACAATTTGTTTTG-CAGGGTGTGCCGGATAGGGTGACAGAT 537

33.12 TAGCAAATCTCAACAGCAGCAATTTGTTTTG-CAGGGTGTGCCGGATAGGGTGACAGAT 537

33.3 TAGCAAATGTTAGCCGCAGCAATTTGTTTTGACAGGGTGTGCCGGATAGGG-GACAGAT 537

33.7 TAGCAAATCTTAGCCGCAGCAATTTGTTTTG-CAGGGTGTGCCGGATAGGGTGACAGAT 538

33.10 TAGCAAATCTTAGCCGCAGCAATTTGTTTTG-CAGGGTGTGCCGGATAGGGTGACAGAT 537

33.1 TAGCAAATCTTAGCCGCAGCAATTTGTTTTG-CAGGGTGTGCCGGATAGGGTGACAGAT 537

33.2 TAGCAAATCTTAGCCGCAGCAATTTGTTTTG-CAGGGTGTGCCGGATAGGGTGACAGAT 537

******** * * * *** ******** *** ******************* *******

**Fig. S15** Multiple sequence alignment using Clustal Omega of *PDS* fragments cloned from the green mutant plant PDS.33g. WT, DNA sequence of the D1 clone isolated from non-transformed ‘Désirée’. The 20-bp region corresponding to the sgRNA is in red and blue colours. The *Mly*I recognition site is in blue. Differences compared to WT are highlighted in green.

**PDS.5g GREEN plant**

WT TTTCCCCGAAGCTTTACCCGCTCCTTTAAATGGTGAGCACATCATAAATAATCATGTTGT 60

5.2 TTTCCCCGAAGCTTTACCCGCTCCTTTAAATGGTGAGCACATCATAAATAATCATGTTGT 60

5.5 TTTCCCCGAAGCTTTACCCGCTCCTTTAAATGGTGAGCACATCATAAATAATCATGTTGT 60

5.1 TTTCCCCGAAGCTTTACCCGCTCCTTTAAATGGTGAGCACATCATAAATAATCATGTTGT 60

5.3 TTTCCCCGAAGCTTTACCCGCTCCTTTAAATGGTGAGCACATCATAAATAATCATGTTGT 60

5.4 TTTCCCCGAAGCTTTACCCGCTCCTTTAAATGGTGAGCACATCATAAATAATCATGTTGT 60

************************************************************

WT CAAACTTCCCTAAAAGTTATTAAATTGATTATTCAATTACACCTATGGGACTTTACTAAC 120

5.2 CAAACTTCCCTAAAAGTTATTAAATTGATTATTCAATTACACCTATGGGACTTTACTAAC 120

5.5 CAAACTTCCCTAAAAGTTATTAAATTGATTATTCAATTACACCTATGGGACTTTACTAAC 120

5.1 CAAACTTCCCTAAAAGTTATTAAATTGATTATTCAATTACACCTATGGGACTTTACTAAC 120

5.3 CAAACTTCCCTAAAAGTTATTAAATTGATTATTCAATTACGCCTATGGGACTTTACTAAC 120

5.4 CAAACTTCCCTAAAAGTTATTAAATTGATTATTCAATTACACCTATGGGACTTTACTAAC 120

**************************************** *******************

WT CTTAAAAGAGCAGTAAGGTTCATTACTTCTTCATCGGACCTTTTGTGTGCAGCTAAAGTG 180

5.2 CTTAAAAGAGCAGTAAGGTTCATTACTTCTTCATCGGACCTTTTGTGTGCAGCTAAAGTG 180

5.5 CTTAAAAGAGCAGTAAGGTTCATTACTTCTTCATCGGACCTTTTGTGTGCAGCTAAAGTG 180

5.1 CTTAAAAGAGCAGTAAGGTTCATTACTTCTTCATCGGACCTTTTGTGTGCAGCTAAAGTG 180

5.3 CTTAAAAGAGCAGTAAGGTTCATTACTTTTTCATCGGACCTTTTGTGTGCAGCTAAAGTG 180

5.4 CTTAAAAGAGCAGTAAGGTTCATTACTTTTTCATCGGACCTTTTGTGTGCAGCTAAAGTG 180

**************************** *******************************

WT TTAACTGCTTTGATATAATTTGCAGGAGTTTTGGCCATCCTAAAGAACAATGAAATGCTT 240

5.2 TTAACTGCTTTGATATAATTTGCAGGAGTTTTGGCCATCCTAAAGAACAATGAAATGCTT 240

5.5 TTAACTGCTTTGATATAATTTGCAGGAGTTTTGGCCATCCTAAAGAACAATGAAATGCTT 240

5.1 TTAACTGCTTTGATATAATTTGCAGGAGTTTTGGCCATCCTAAAGAACAATGAAATGCTT 240

5.3 TTAACTGCTTTGATATAATTTGCAGGAGTTTTGGCCATCCTAAAGAACAATGAAATGCTT 240

5.4 TTAACTGCTTTGATATAATTTGCAGGAGTTTTGGCCATCCTAAAGAACAATGAAATGCTT 240

************************************************************

WT ACATGGCCAGAGAAAGTCAAATTTGCAATTGGACTCTTGCCAGCAATGCTTGGAGGGCAA 300

5.2 ACATGGCCAGAGAAAGTCAAATTTGCAATTGGACTCTTGCCAGCAATGCTTGGAGGGCAA 300

5.5 ACATGGCCAGAGAAAGTCAAATTTGCAATTGGACTCTTGCCAGCAATGCTTGGAGGGCAA 300

5.1 ACATGGCCAGAGAAAGTCAAATTTGCAATTGGACTCTTGCCAGCAATGCTTGGAGGGCAA 300

5.3 ACATGGCCAGAGAAAGTCAAATTTGCAAT**C**--ACTCTTGCCAGCAATGCTTGGAGGGCAA 300

5.4 ACATGGCCAGAGAAAGTCAAATTTGCAATTGGACTCTTGCCAGCAATGCTTGGAGGGCAA 300

***************************** ******************************

WT TCTTATGTTGGGGCTCAAGATGGGATAAGTGTCAAGGACTGGATGAGAAAGCAAGTGCGT 360

5.2 TCTTATGTTGAGGCTCAAGATGGGATAAGTGTCAAGGACTGGATGAGAAAGCAAGTGCGT 360

5.5 TCTTATGTTGAAGCTCAAGACGGGATAAGTGTTAAGGACTGGATGAGAAAGCAAGTGCGT 360

5.1 TCTTATGTTGAAGCTCAAGACGGGATAAGTGTTAAGGACTGGATGAGAAAGCAAGTGCGT 360

5.3 TCTTATGTTGAAGCTCAAGACGGGATAAGTGTTAAGGACTGGATGAGAAAGCAAGTGCGT 360

5.4 TCTTATGTTGAAGCTCAAGACGGGATAAGTGTTAAGGACTGGATGAGAAAGCAAGTGCGT 360

********** ******** *********** ***************************

WT AAACAGTTATGTTACTTTTTAAGCATAAACCTGGCTGTATAGTTCACAGTTCACACACAA 420

5.2 AATCAGTTATGTTACTTTTTAAGCATAAACCTGGCTGTATAGTTCACAGTCCACACACAA 420

5.5 AATTAGTTATGTTACTTTTTAAGCATAAACCTGGCTGTATTGTTCACAGTTCACACACAA 420

5.1 AATTAGTTATGTTACTTTTTAAGCATAAACCTGGCTGTATTGTTCACAGTTCACACACAA 420

5.3 AATTAGTTATGTTACTTTTTAAGCATAAACCTGGCTGTATAGTTCACAGTTCACAC--AC 418

5.4 AATTAGTTATGTTACTTTTTAAGCATAAACCTGGCTGTATAGTTCACAGTTCACAC--AC 418

** ************************************ ********* ***** *

WT ATTGTTCATTTGAGGTGTTAGATTGTCTACCTGCCTTTCTTTTGCTAACAGCATACTTCT 480

5.2 ATTGTTCATTTGAGGTGTTAGATTGTCTACCTGCCTTTCTTTTGCTAACAGCATACTTCT 480

5.5 ATTGTTCATTTGAGGTGTTAGATTGTCTACCTGCCTTTCTTTTGCTAGCCGCATACATCT 480

5.1 ATTGTTCATTTGAGGTGTTAGATTGTCTACCTGCCTTTCTTTTGCTAGCCGCATACATCT 480

5.3 ATTGTTCATTTGAGGTGTTAGATTGTCTACCTGCCTTTCTTTTGCTAGCAGCATACATCT 478

5.4 ATTGTTCATTTGAGGTGTTAGATTGTCTACCTGCCTTTCTTTTGCTAGCCGCATACATCT 478

*********************************************** * ****** ***

WT AGCAAATCTCAGCAGCAGCAATTTGTTTTGCAGGGTGTGCCGGATAGGGTGACAGAT 537

5.2 AGCAAATCTCAGCAGCAGCAATTTGTTTTGCAGGGTGTGCCGGATAGGGTGACAGAT 537

5.5 AGCAAATCTTAGCCGCAGCAATTTGTTTTGCAGGGTGTGCCGGATAGGGTGACAGAT 537

5.1 AGCAAATCTTAGCCGCAGCAATTTGTTTTGCAGGGTGTGCCGGATAGGGTGACAGAT 537

5.3 AGCAAATCTTAGCCGCAGCAATTTGTTTTGCAGGGTGTGCCGGATAGGGTGACAGAT 535

5.4 AGCAAATCTTAGCCGCAGCAATTTGTTTTGCAGGGTGTGCCGGATAGGGTGACAGAT 535

********* *** *******************************************

**Fig. S16** Multiple sequence alignment using Clustal Omega of *PDS* fragments cloned from the green mutant plant PDS.5g. WT, DNA sequence of the D1 clone isolated from non-transformed ‘Désirée’. The 20-bp region corresponding to the sgRNA is in red and blue colours. The *Mly*I recognition site is in blue. Differences compared to WT are highlighted in green.
