# Supplementary material for: Generation of transgene-free PDS mutants in potato by Agrobacterium-mediated transformation
Source: BMC Biotechnol. 2020 May 12;20:25. doi: 10.1186/s12896-020-00621-2 (PMC7216596; doi:10.1186/s12896-020-00621-2)
Supplement: Supplementary file 3 — Additional file 3: Figure S1. Detection and expression of Cas9 in PDS mutants obtained after continuous Km selection. Figure S2. Detection of the lack of transgenes in PDS mutants obtained after 3 days of Km selection. [file 12896_2020_621_MOESM3_ESM.docx]

**Fig. S2** PCR and RT-PCR analysis of *PDS* mutants generated by continuous Km selection. Lanes 1-7, *PDS* mutants; #3 is “tabby”, #7 does not carry Cas9, however, is resistant for Km; D, Désirée. *ACTIN* was tested as a cDNA quantity control. The *Cas9* fragment was generated using the Cas9-1133 Fw + Cas-1462 R primer combination, while the *ACTIN* fragment was obtained with the Actin Fw + Actin R primer pair. Primer sequences are listed in Additional file 1: Table S1.

**Fig. S3** PCR analysis of *PDS* mutant plants generated by 3-day Km selection. Lanes 1-5, *PDS* mutant plants; D, Désirée; P, PROGED::gPDS. PCR analysis was carried out with primers specific for the *Cas9-scfRNA* (Cas9-1594 Fw + PacScfRNA R), *Cas9* (Cas9-59 Fw + Cas9-2019 R), *nptII* (npt II PROGMO Fw + npt II PROGMO R) and *PDS* (PDS Fw + PDS R). *PDS* was tested as a quantity control of plant genomic DNA. Primer sequences are listed in Additional file 1: Table S1. From plant DNA 200 ng, while from plasmid DNA 5 ng was used in PCR.
